# Development of a prognostic model for hepatocellular carcinoma based on microvascular invasion characteristic genes by spatial transcriptomics sequencing

**DOI:** 10.3389/fimmu.2025.1529569

**Published:** 2025-02-20

**Authors:** Xiaolan Mu, Lili Pan, Xicheng Wang, Changcheng Liu, Yu Li, Yongchao Cai, Zhiying He

**Affiliations:** ^1^ Institute for Regenerative Medicine, Medical Innovation Center and State Key Laboratory of Cardiology, Shanghai East Hospital, School of Life Sciences and Technology, Tongji University, Shanghai, China; ^2^ Shanghai Engineering Research Center of Stem Cells Translational Medicine, Science and Technology Commission of Shanghai Municipality, Shanghai, China; ^3^ Shanghai Institute of Stem Cell Research and Clinical Translation, Shanghai Municipal Education Commission, Shanghai, China

**Keywords:** hepatocellular carcinoma, microvascular invasion, spatial transcriptome sequencing, single-nucleus RNA sequencing, prognostic model

## Abstract

Microvascular invasion (MVI) is an independent risk factor for the recurrence and metastasis of hepatocellular carcinoma (HCC), associated with poor prognosis. Thus, MVI has significant clinical value for the treatment selection and prognosis assessment of patients with HCC. However, there is no reliable and precise method for assessing the postoperative prognosis of MVI patients. This study aimed to develop a new HCC prognosis prediction model based on MVI characteristic genes through spatial transcriptomics sequencing, distinguishing between high-risk and low-risk patients and evaluating patient prognosis. In this study, four MVI samples with different grades were selected for spatial transcriptomic sequencing to screen for MVI region-specific genes. On this basis, an HCC prognostic model was constructed using univariate Cox regression analysis, LASSO regression analysis, random survival forest, and stepwise multivariate Cox regression analysis methods. We constructed a 7-gene prognostic model based on MVI characteristic genes and demonstrated its applicability for predicting the prognosis of HCC patients in three external validation cohorts. Furthermore, our model showed superior predictive performance compared with three published HCC prediction prognostic models and could serve as an independent prognostic factor for HCC. Additionally, single nucleus RNA sequencing analysis and multiple immunofluorescence images revealed an increased proportion of macrophages in high-risk patient samples, suggesting that HCC tumor cells may promote HCC metastasis through MIF-CD74 cell interactions. To sum up, we have developed a 7-gene biomarker based on MVI that can predict the survival rate of HCC patients at different stages. This predictive model can be used to categorize into high- and low- risk groups, which is of great significance for the prognostic assessment and personalized treatment of HCC patients.

## 1 Introduction

Hepatocellular carcinoma (HCC) is the primary liver cancer accounts for approximately 85 to 90% of all liver cancers (1). It has an insidious onset, and easy recurrence and metastasis. This makes HCC the sixth most common cancer and the third leading cause of cancer-related death (2). Surgical resection, liver transplantation, neoadjuvant therapy and targeted drugs are the main methods for early to intermediate stage HCC (3–5). Although these methods have improved the effectiveness of HCC treatment, over 70% of patients experience recurrence within 5 years after surgery, indicating a poor prognosis (6). Ninety percent of cases of recurrence and death are related to metastases (7). Therefore, there is an urgent need to develop new prognostic assessment methods to predict the clinical prognosis of HCC patients. Constructing prognostic models to predict survival rates and classify patients remains of great significant importance.

Tumor cells infiltrate blood vessels and form vascular cancer thrombi during the metastasis process. Microvascular invasion (MVI) refers to the presence of cancer cell nests in the lumens of blood vessels lined with endothelium under a microscope (8, 9). MVI represents an early stage of vascular infiltration and metastasis in HCC and is an independent prognostic factor for tumor recurrence and metastasis in HCC patients (10, 11). The prediction of HCC prognosis is vital for the selection of therapeutic approaches and prognostic improvement in patients with HCC. Consequently, more accurate predictive markers of MVI are needed to evaluate the risk of tumor recurrence and the prognosis of HCC patients. The tumor microenvironment (TME) plays an important role in the formation of MVI. However, conventional sequencing methods have difficulty analyzing the differential genes, microenvironmental changes and cellular heterogeneity in the MVI sites of HCC. Single-cell RNA sequencing (scRNA-seq) can reveal variations between different types and cell heterogeneity. This technology is widely used in various cancer studies, including studies of liver (12, 13), breast (14), and kidney cancer (15). Single-nucleus RNA sequencing (snRNA-seq) can also classify cells and map the cellular atlas of tissues. However, because single-cell RNA sequencing is only suitable for fresh tissue, many clinical frozen clinical samples cannot be subjected to single-cell RNA sequencing (16). Moreover, the dissociation process in single-cell RNA sequencing induces the expression of stress genes, leading to transcription biases in cells (17, 18). Furthermore, studies have shown that snRNA-seq works consistently with scRNA-seq and accurately captures the transcriptional state of cells, which has been confirmed in various tissues (16, 19, 20). Therefore, this study employs single-nucleus RNA sequencing instead of single-cell RNA sequencing. Nevertheless, single-nucleus sequencing loses spatial location information during the nucleus isolation process, making it difficult to obtain the spatial positioning of individual cells within tissues. The recent development of spatial transcriptomics (ST) has enabled the sequencing of smaller tissue samples to obtain gene expression profiles of specific locations and spatial locations of cells. Spatial transcriptomics generates complete transcriptome data from an entire tissue sample and allows the localization and differentiation of functional genes in specific tissue regions, creating spatial expression maps of cells and genes (21). This technology is now widely used in the research of various diseases, including gene expression and cell mapping during heart development (22), pancreatic cancer (23), prostate cancer (24), and skin squamous cell carcinoma (25). It is currently assumed that MVI is located mainly at the junction between the tumor and adjacent tumor. ST can obtain not only transcriptome data of the connection between the tumor and adjacent tumor but also data of the MVI region, and directly screen the characteristic genes of the MVI region. In conclusion, we combined spatial transcriptome sequencing and single-nucleus RNA sequencing techniques to obtain differentially expressed genes and determine the microenvironment composition of microvascular invasion sites. These finding are crucial for understanding the mechanism of MVI formation and finding new treatment targets.

The purpose of this study was to investigate MVI molecular markers using spatial transcriptome technology and to construct a prognostic risk assessment model for HCC patients based on MVI, with the aim of providing appropriate treatment methods for HCC patients.

## 2 Materials and methods

### 2.1 Human HCC tissues

From May 2020 to February 2021, a total of 28 early HCC tumors and adjacent normal tissues were collected from patients who underwent surgical resection at the Eastern Hepatobiliary Surgery Hospital (Shanghai, China). Each tissue sample was approximately 1 cm ×1 cm × 1 cm in size, washed in PBS, dehydrated, and quickly frozen in isopentane and liquid nitrogen. The samples were subsequently transported to the laboratory on dry ice. The tissues were embedded in optimal cutting temperature (OCT) compound (Sakura, catalog no. 4583) and stored at -80°C until use. The cryosections were then subjected to H&E staining to determine the number and distribution of MVI. The samples were sent to OE Biotech for spatial transcriptomics and single nucleus-RNA sequencing. All diagnoses were examined histologically by a specialized pathologist.

### 2.2 Spatial transcriptomics sequencing

This experiment utilized the 10x Genomics Visium technology platform. All reagents and consumables used in the experiment were provided by this platform. Detailed product numbers are available at www.10xgenomics.com/products/spatial-gene-expression. After fixation, H&E staining, and imaging of the sections, tissue-specific permeabilization was performed using kits provided by 10x. Library construction and sequencing were then performed using spatially barcoded mRNA-binding oligonucleotides according to standard protocols of the 10x Genomics platform.

### 2.3 Spatial transcriptome sequencing analysis

After the raw spatial transcriptomics data were obtained, Space Ranger was used for data quality control. The generated spot matrices were analyzed using the “Seurat7” package. Subsequently, we utilized principal component analysis (PCA) to reduce dimensions, t-distributed Stochastic Neighbor Embedding (t-SNE) to demonstrate clusters, and the mutual nearest neighbors (MNN) algorithm to eliminate batch effects. Next, genes with spatial expression patterns were identified using the FindMarkers function, followed by Gene Ontology (GO) enrichment and Kyoto Encyclopedia of Genes and Genomes (KEGG) pathway analyzes for the differentially expressed genes.

### 2.4 Single-nucleus RNA sequencing

The frozen liver tissue was rinsed twice with medium, and then the frozen liver tissue was minced. Then resuspend the minced liver tissue in 0.5 mL ice-cold EZ lysis buffer and homogenize on ice. The homogenized liver tissue is then successively filtered through a cell strainer. Next, centrifuge the filtered liver tissue for 5 min at 4°C and 500 g to precipitate the cell nucleus. Subsequently, resuspend the precipitated cell nucleus in 1 mL of ice-cold buffer and filter through a 20 μm cell strainer. Finally, proceed immediately to single-nucleus RNA sequencing of the obtained the cell nucleus.

### 2.5 Single-nucleus RNA sequencing analysis

After obtaining the raw single nucleus data, we first utilized Cell Ranger for data quality control and gene qualification. Following quantification, we filtered out low quality cells and low abundance genes. Subsequently, we applied MNN and t-SNE algorithms for dimensionality reduction and clustering. Then we annotated the cell types using the “SingleR” package and our own statistically determined specific marker genes. Finally, we selected differentially expressed genes based on the fold change and *p*-value results, and performed GO enrichment analysis and KEGG pathway enrichment analysis for these genes.

### 2.6 Data acquisition

A total of 424 TCGA-LIHC transcriptome sequencing datasets, 371 single nucleotide variation (SNV) datasets, and 377 clinical information datasets were downloaded from The Cancer Genome Atlas (TCGA) database (https://portal.gdc.cance.gov/). After the data were integrated, 371 primary HCC transcriptome sequencing data, 171 early Tumor Node Metastasis classification (TNM) HCC transcriptome sequencing data, and 167 early TNM HCC SNV data were obtained. In addition, 225 cases of HCC microarray data and survival information were retrieved from the GSE14520 dataset in the Gene Expression Omnibus (GEO) database (https://www.ncbi.nlm.nih.gov/geo/), including 93 cases of early-stage TNM HCC microarray data; and 115 cases of HCC microarray data and survival information were obtained from the GSE76427 dataset. From the International Cancer Genome Consortium (ICGC) LIRI-JP dataset (https://dc.icgc.org/), 240 cases of HCC transcriptomic sequencing data and survival information were collected. The TCGA dataset acted as a training set for building the predictive prognosis model, while the GSE14520, GSE76427 and LIRI-JP datasets served as validation sets for external validation of the model.

### 2.7 Gene set variation analysis

The HALLMARK gene sets were collected from the MSigDB database (Molecular Signature Database, http://www.gsea-msigdb.org/gsea/msigdb). Gene set variation analysis (GSVA) was employed to assess HALLMARK pathway scores in HCC patients. Pearson correlation coefficient was utilized to examine the relationships between risk scores and HALLMARK signaling pathways. A *p* value < 0.05 was considered statistically significant.

### 2.8 Evaluation and validation of the prognostic models

The “Survminer” package was used to identify the optimal risk score cutoff and calculate risk scores for HCC patients. Patients were divided into high and low risk groups according to the best cutoff value. The Kaplan-Meier survival curves show the prognosis of the high and low risk groups and the log-rank test evaluate survival differences between the two groups. The “timeROC” package was used to draw 1-year, 2-year, and 3-year Receiver Operating Characteristic (ROC) curves and calculate the Area Under the curve (AUC). The ROC and AUC curve can be used to estimate the diagnostic value of the prognostic model in predicting the prognosis of HCC patients. The survival analysis was conducted in the GSE14520 external validation cohort, and the ROC curves were plotted to verify the stability and accuracy of the prognostic model. To further assess the predictive performance of the prognostic model, we calculated the risk scores of liver cancer patients at all stages in the TCGA, GSE14520, GSE76427 and LIRI-JP cohorts and performed survival analysis for each group. The “AUCell” package was used to evaluate the expression of the prognostic model gene set in each region of the spatial transcriptome. The ssGSEA algorithm of the “GSVA” package was used to estimate the expression of the prognostic gene set in each cell type from single-nucleus transcriptome sequencing.

### 2.9 This model is compared with three published MVI-related models

This model was compared with three existing MVI-related models. Scores for HCC patients were calculated using scoring formulas provided in two publications, grouped by optimal cutoff values for survival analysis, and ROC curves were plotted. The correlation between this model and the three existing MVI-related models was examined using Pearson correlation analysis, with *p* < 0.05 considered statistically significant.

### 2.10 Clinical characteristics of the signature

To determine the correlation between the risk score and clinical characteristics (age, gender, Grade classification, ChildPugh classification, alcohol consumption, hepatitis B), we applied the Wilcoxon test for assessment. ROC curves of the prognostic model and various clinical characteristics were plotted using the “pROC” package, and the AUC and concordance index values were compared. To determine whether the prognostic model is a prognostic factor for HCC patients, multivariate Cox regression analysis was performed on the prognostic model, age, gender, Grade classification, ChildPugh classification, alcohol consumption, Hepatitis B virus (HBV), Hepatitis C virus (HCV), alpha-fetoprotein, platelet count, prothrombin, albumin, and creatinine levels to identify prognostic factors for HCC. Nomograms for prognostic factors in HCC patients were plotted using the “rms” package, with each patient assigned points for each prognostic factor. The sum of these values resulted in a total score that was used to predict the 1-year, 3-year, and 5-year survival rates of patients with HCC. To compare the predicted survival rates with those observed and to evaluate the accuracy of the nomogram, a 5-year calibration curve was constructed.

### 2.11 Characteristics of different risk groups

The “DESeq2” package was used to identify genes highly expressed in the high-risk group with a fold change (FC) > 2 and an adjusted p value < 0.05. These highly expressed genes were subjected to KEGG pathway enrichment analysis, and pathways with a *p* value < 0.05 were considered enriched. Waterfall plots for ten most frequently mutated genes in both the high- and low-risk groups were created using the oncoplot function from the “maftools” package. The tumor mutation burden (TMB) for each patient was computed and a Pearson correlation analysis was performed between the risk score and the TMB, with a correlation coefficient and a p value < 0.05 considered statistically significant. The Tumor Immune Estimation Resource (TIMER) 2.0 database (http://timer.cistrome.org/) can assess the infiltration of six types of immune cells in TCGA, including B cells, CD4+ T cells, CD8+ T cells, neutrophils, macrophages, and dendritic cells. The Tumor Immune Dysfunction and Exclusion (TIDE) score and exclusion score are calculated via TIDE (http://tide.dfci.harvard.edu/) to predict and the immune escape ability of HCC and infer the effectiveness of immunotherapy in HCC patients.

### 2.12 Cell interaction analysis

Malignant cell scores in MVI samples were calculated using the ssGSEA algorithm. Malignant cells are categorized into high-score and low-score groups based on the median of these scores. The “CellChat” package was used to analyze cell the interactions between high-score and low-score malignant cells and other cell types to calculate and infer the cell interaction networks. The number of interactions, the strength of interactions, and the ability to send and receive signals are compared between high-score and low-score malignant cells.

### 2.13 Quantitative real-time PCR

Total RNA was isolated from liver samples using TRIzol^®^ LS Reagent (Thermo Scientific, USA), and 250 µl of fluid was added to 750 µl TRIzol LS. Subsequently, 200 µl of chloroform was used for phase separation and 100% isopropanol and Glycogen (Beyotime, Shanghai, China) were used for RNA precipitation. Finally, the RNA was eluted in 10 µl RNase-free water after being washed twice in 75% ethanol. cDNA was synthesized by reverse transcription with a PrimeScript™ RT Master Mix (Takara, Japan). qPCR was performed using 2x SYBR Green qPCR Master Mix (bimake, USA) with ABI Prism Q7 System (Thermo Fisher Scientific, USA) in a 10 µl reaction system. Expression of different genes were normalized to GAPDH and were analyzed using the 2-ΔΔCT method. The primers used in this study are shown in **Supplementary Table S1**.

### 2.14 Gene set variation analysis

GSVA is a non-parametric and unsupervised approach for assessing the enrichment of transcriptome gene sets. It evaluates the enrichment of metabolic pathways in samples by synthesizing scores for the gene sets of interest, transforming gene-level variations into pathway-level changes to infer the biological functions of samples. In this study, we subclassify myeloid cells and use the GSVA algorithm to comprehensively score macrophages and non-macrophages within myeloid cells, thereby assessing the potential biological function changes in macrophages and non-macrophages.

### 2.15 Western blotting

The HCC tissues were lysed using RIPA buffer supplemented with a protease inhibitor cocktail. The protein samples were then resolved using SDS-PAGE and transferred to PVDF membranes (Millipore, no. ISEQ00010). After blocking the membranes with 5% skimmed milk (in TBST) for 1h at room temperature, they were incubated with the primary antibody overnight at 4°C. Subsequently, the membranes were incubated with the HRP-conjugated IgG at room temperature for 1h. Finally, the bands were visualized using enhanced chemiluminescence. Antibodies used are listed as follow: GAPDH (Proteintech, Cat No. 10494, 1:5000), SPLI (Abclonal, Cat No. A1897, 1:1000), GPX2(Abclonal, Cat No. A15999, 1:1000), CFL1 (Proteintech, Cat No. 10960, 1:3000), CANX (Proteintech, Cat No. 10427, 1:5000), DCN (Proteintech, Cat No. 14667, 1:2000), CARHSP1 (Proteintech, Cat No. 11672, 1:1500), PIGO (Abclonal, A18670, 1:1000).

### 2.16 Multiple immunofluorescence

Tissue paraffin sections were baked at 60 °C for 1 hour, and then placed in xylene I/II for 15 minutes for dewaxing. Different alcohol concentrations (95%, 80%, 70%, 50%) were used for hydration. The citrate antigen retrieval solution (PH 6.0) (MXB, China) was carried out in the microwave for 20 minutes. Endogenous peroxidase was blocked with 3% H_2_O_2_ at room temperature for 15 minutes in the dark. Blocking was performed with 3% BSA. The primary antibody was incubated overnight at 4°C. The next day, the primary antibody was washed off with PBST, the HRP-conjugated secondary antibody was added for 50 minutes at room temperature. A ready-to-use fluorescent dye was added and incubated for 10 minutes at room temperature (Abclonal; China). The antibody was washed, repeating the steps with 3% H_2_O_2_ until staining with the three primary antibodies was completed. DAPI incubation for 10 min was carried out for nuclear counterstaining, followed by slide sealing and microscopic examination. MIF (Proteintech, USA, 1:250); CD68 (CST, USA, 1:2000); CD74 (Santa Cruz; USA, 1:250).

### 2.17 Statistical analysis

In this study, GraphPad Prism 8.0 and R software v4.0.1 were used for the statistical analysis and plotting of the experimental data. A *p* < 0.05 was considered statistically significant.

## 3 Results

### 3.1 Identification of differentially expressed genes in MVI by spatial transcriptome analysis

**Figure 1:**
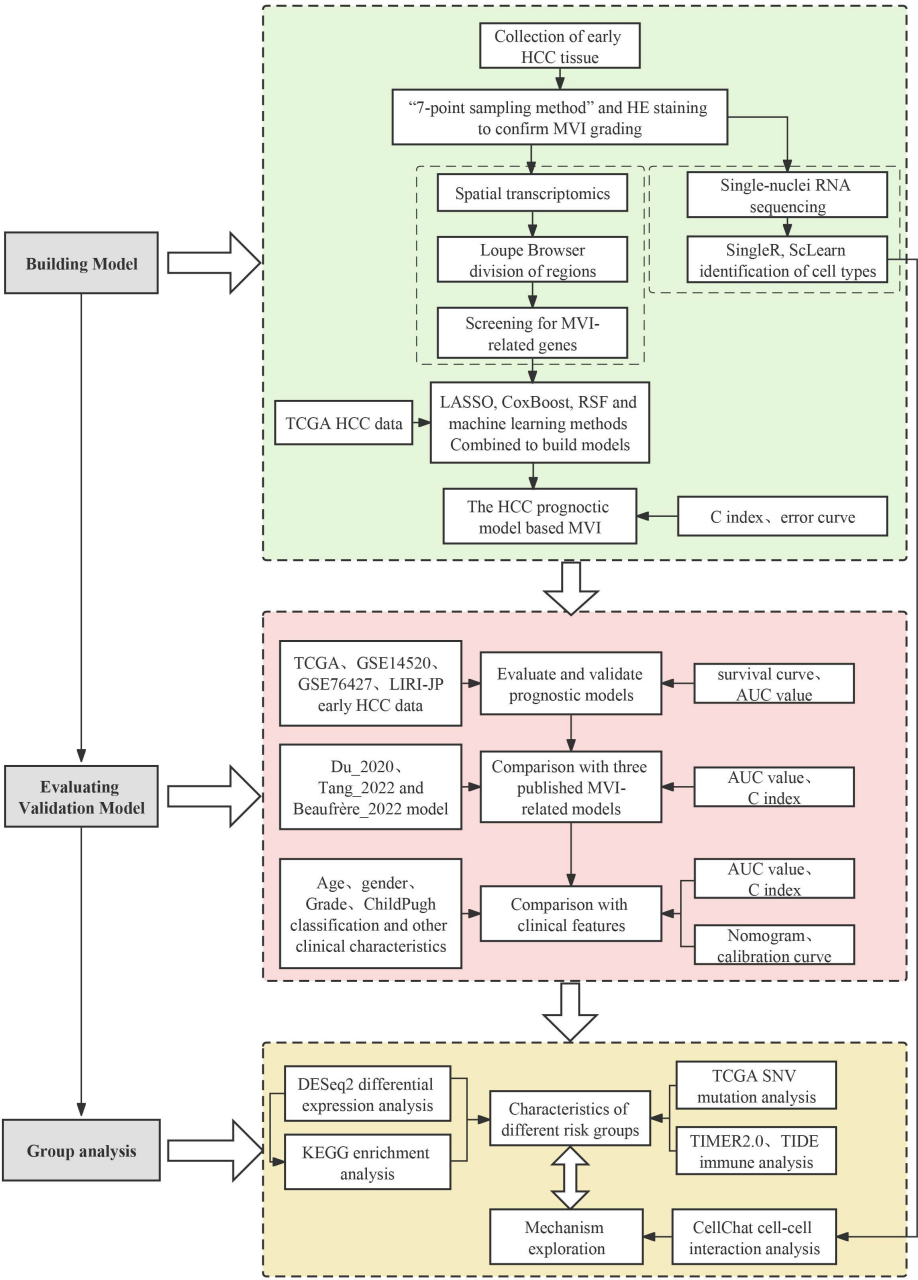
Workflow of this study.

**Figure 2:**
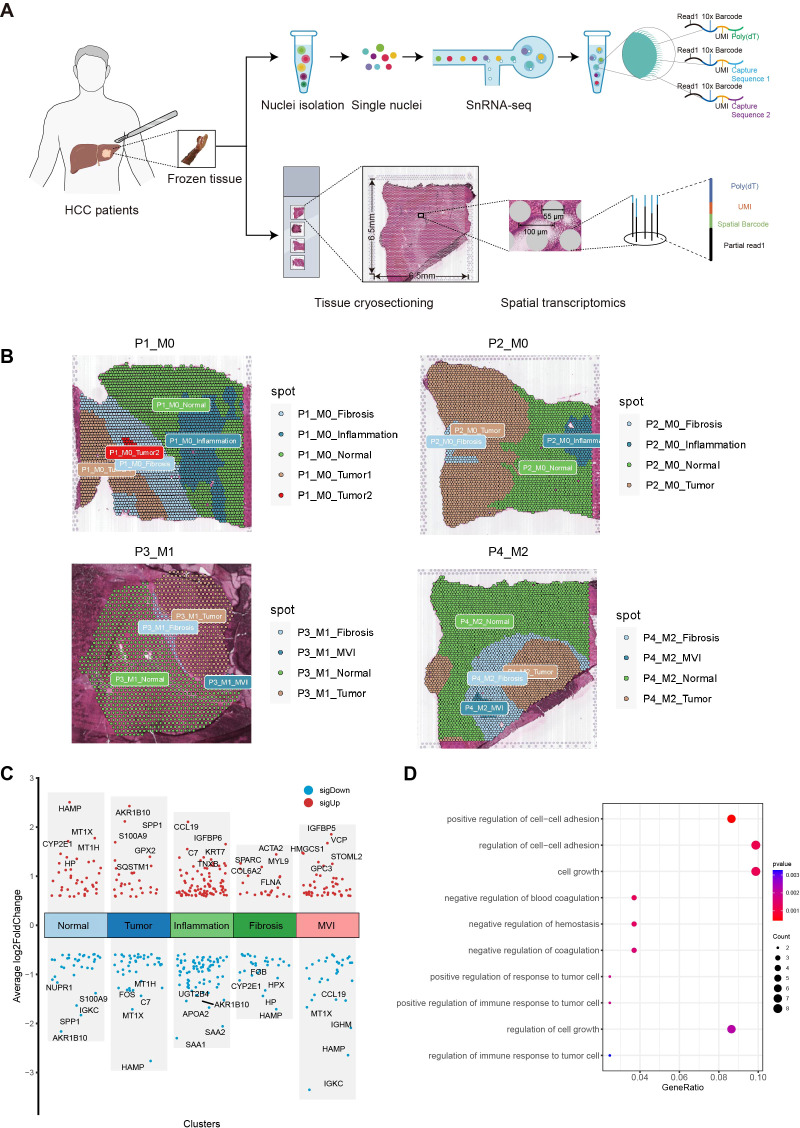
Exploration of MVI differential genes with ST. **(A)** Workflow of Hepatocellular carcinoma samples collection, processing for spatial transcriptomics sequencing, single-nuclei RNA sequencing and data analysis. **(B)** Regional division of HCC tissue sections: Tumor, Normal, MVI, Inflammation and fibrosis areas. **(C)** Plot of differential genes in five regions delineated by spatial transcriptome sequencing. Red dots indicate genes up-regulated in the five regions. Blue points indicate genes down-regulated in the five regions. **(D)** Gene Ontology enrichment analysis of MVI differential genes. MVI, Microvascular Invasion; HCC, Hepatocellular Carcinoma; GO, Gene Ontology.

To understand the causes of microvascular invasion in HCC and identify new biomarkers, we employed spatial transcriptome sequencing to discover novel targets. The workflow of this study is shown in **Figure 1**. We collected 25 pairs of early HCC patient tumors and adjacent normal tissues for cryo-embedding, and the MVI grade and quantity were determined by H&E staining. Complete clinical and pathological information can be found in the **Supplementary Table S2**. To analyze the differentially expressed genes in the MVI regions of hepatocellular carcinoma patients, we performed spatial transcriptome sequencing on 2 M0 and 2 MVI samples (P1_M0, P2_M0, P3_M1, P4_M2) (**Supplementary Figure 1A**). The spatial transcriptome technology in this study utilized the 10x Genomics Visium platform with spot diameters of 55 μm (containing 8-20 cells) (**Figure 2A**), and the 6.5 mm × 6.5 mm capture area contained 5000 spots. In this study, Space Ranger was used to assess the quality of the spatial transcriptome sequencing data, yielding a total of 13546 spots. After subsequent quality control and batch effect correction, 11620 spots remained (**Supplementary Figures 1B, C**). Furthermore, the data showed that the number of spots per sample was approximately 3000, with an average gene number per spot of approximately 3782 and an average Unique Molecular Identifier (UMI) number per spot of 15157 (**Supplementary Table S3**). Overall, UMI and gene counts were higher in tumor regions than in normal areas, which is consistent with previous studies (**Supplementary Figures 1B, C**).

Tissue sections were segmented by pathologists from our hospital into five different regions: tumor, normal, inflammation, MVI and fibrosis areas (**Figure 2B**). To verify whether the transcriptomic features matched the histological information, we compared H&E images with the corresponding spatial transcriptome data. The results confirmed that the regions defined by the expression of cell type marker genes were highly consistent with their pathological images. Specifically, ALB and CYP2E1 were highly expressed in normal areas, GPC3 and AKR1B10 were highly expressed in tumor areas, ACTA2 and COL1A1 were highly expressed in fibrostic areas, and PTPRC was highly expressed in inflammation areas (**Supplementary Figure 2**).

Next, we performed a differential expression analysis for the five regions using the FindAllMarkers function, with the criteria of absolute fold change (|FC|) > 1.5 and *p.adj* < 0.05. *A* total of 82 potential MVI-related genes were identified, including 49 upregulated genes and 33 downregulated genes (**Figure 2C**). GO enrichment analysis revealed that these genes are involved in biological processes such as the regulation of intercellular adhesion, cell growth, coagulation, and the regulation of the immune response in tumor cells (**Figure 2D**). These 82 differentially expressed genes were identified as MVI-related and will be used for subsequent modeling.

### 3.2 Construction of the HCC prognostic model on the basis MVI characteristic genes

We employed various analysis methods, including univariate Cox regression analysis, LASSO regression analysis, multivariate Cox regression analysis, CoxBoost, random survival forest, and stepwise regression analysis, to select the optimal HCC prediction model in the TCGA training cohort (**Supplementary Table S4**).

Univariate Cox regression analysis was conducted on the early TCGA HCC dataset to identify MVI genes associated with patient prognosis. A total of 13 MVI genes related to the prognosis of HCC patients were selected. These 13 MVI genes were then analyzed using LASSO regression, resulting in 8 genes with non-zero coefficients (**Supplementary Figures 3A, B**). Finally, a bidirectional stepwise multivariate Cox regression was performed for these 8 genes to obtain the best prognostic model based on the lowest AIC value.

CoxBoost was used to find the best model fit when the optimal boosting step was performed as 97 through 10-fold cross-validation, and picked out six non-zero coefficients of MVI-related genes were selected (**Supplementary Figures 3C, D**). Then, these 6 genes were subjected to multifactorial Cox regression analysis and bidirectional stepwise regression analysis to optimize this model, obtaining the best model based on the minimal AIC value.

Ultimately, the randomForestSRC package was utilized to perform random survival forest analysis. The error rate was lowest when the random survival forest model included 8 genes (**Supplementary Figures 3E–G**). These 8 genes were then subjected to multifactorial Cox regression analysis and bidirectional stepwise regression analysis to optimize the model, with the best model identified based on the lowest AIC value.

**Figure 3:**
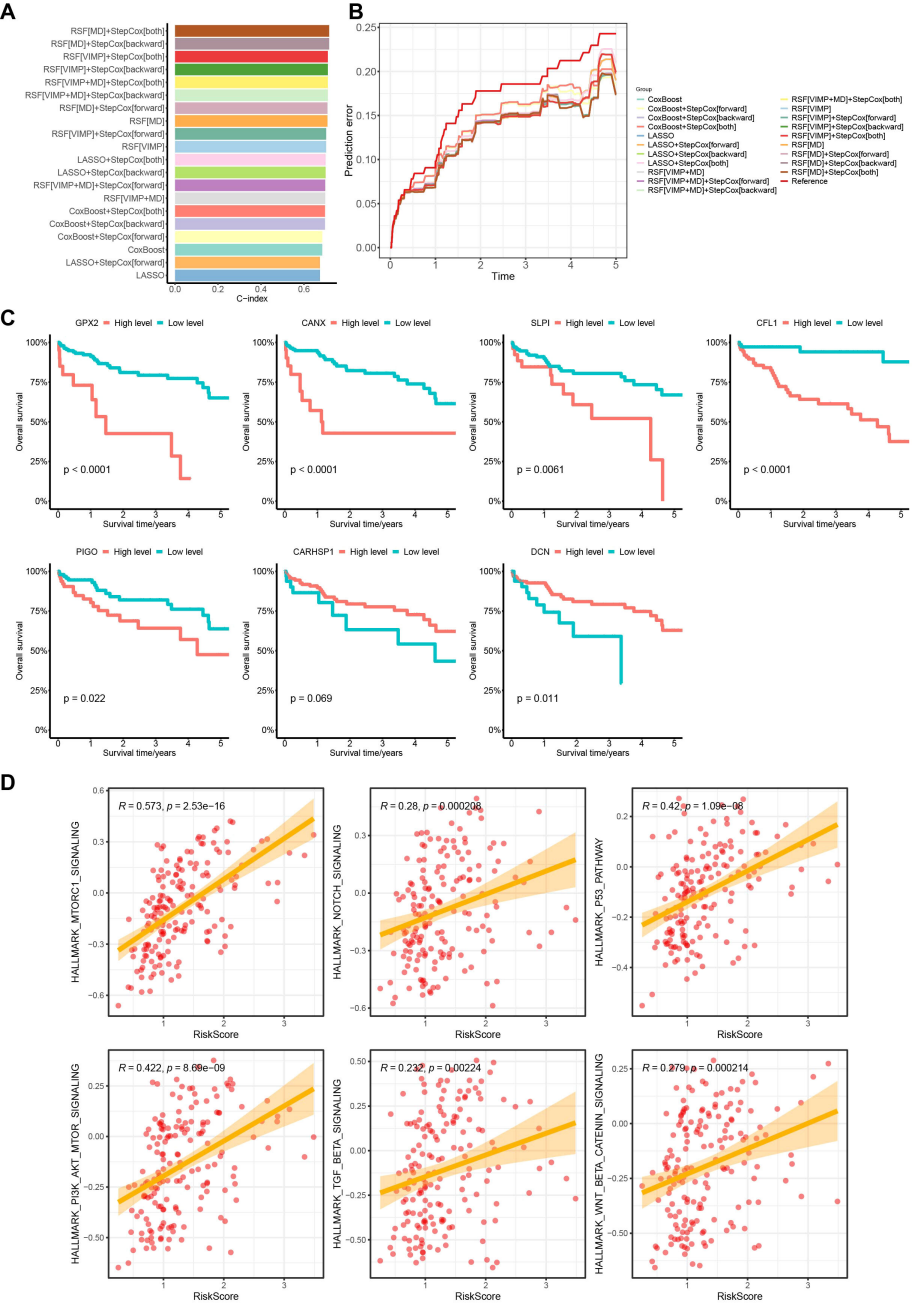
Random survival forest and bidirectional stepwise multifactorial Cox regression identification of key MVI differential genes of the model and prognostic analysis in the TCGA cohort. **(A)** C-indexes of 20 prognostic models obtained by different modeling methods. **(B)** Error curves of 20 prognostic models obtained by different modeling methods. **(C)** Survival analysis of 7 genes with the prognostic model based on the TCGA database. **(D)** Correlation analysis between early HCC patients risk scores and HALLMARK pathways. TCGA, The Cancer Genome Atlas.

The comparison revealed that the model built using a combination of random survival forest and bidirectional stepwise multifactorial Cox regression analysis achieved the highest calibration C-index (0.717107) and the lowest error rate, suggesting that the model created using the combination method has greater prognostic prediction accuracy (**Figures 3A, B**). We then selected 7 key MVI-related genes (GPX2, CANX, SLPI, CFL1, PIGO, CARHSP1, DCN) to construct the HCC prognostic model. The formula for the prognostic risk score was as follows: Risk Score = (0.000376 × GPX2 expression) + (0.002959 × CANX expression) + (0.000203 × SLPI expression) + (0.0045 × CFL1 expression) + (0.056461 × PIGO expression) – (0.026806 × CARHSP1 expression) – (0.0101 × DCN expression). In this model, GPX2, CANX, SLPI, CFL1, and PIGO have positive coefficients and are considered risk-related genes, whereas DCN and CARHSP1 have negative coefficients and are protective genes.

We subsequently plotted Kaplan-Meier curves based on the expression levels of the 7 MVI genes. The curves showed that high expression levels of GPX2, CANX, SLPI, CFL1 and PIGO in patients were significantly associated with lower overall survival than those in low expression groups, which correlated with worse survival rates in HCC patients. DCN expression was associated with better survival rates, whereas high expression of CARHSP1 suggested a better prognosis for HCC patients, although the difference was not statistically significant (**Figure 3C**). We also found that the risk score was positively correlated with HCC-related HALLMARK signaling pathways such as mTORC1, PI3K/AKT/mTOR, and p53, suggesting that the poor prognosis of patients may be the result of a combination of multiple oncogenic pathways (**Figure 3D**).

### 3.3 The risk score based on MVI-related genes might be an independent risk factor for patients with HCC

**Figure 4:**
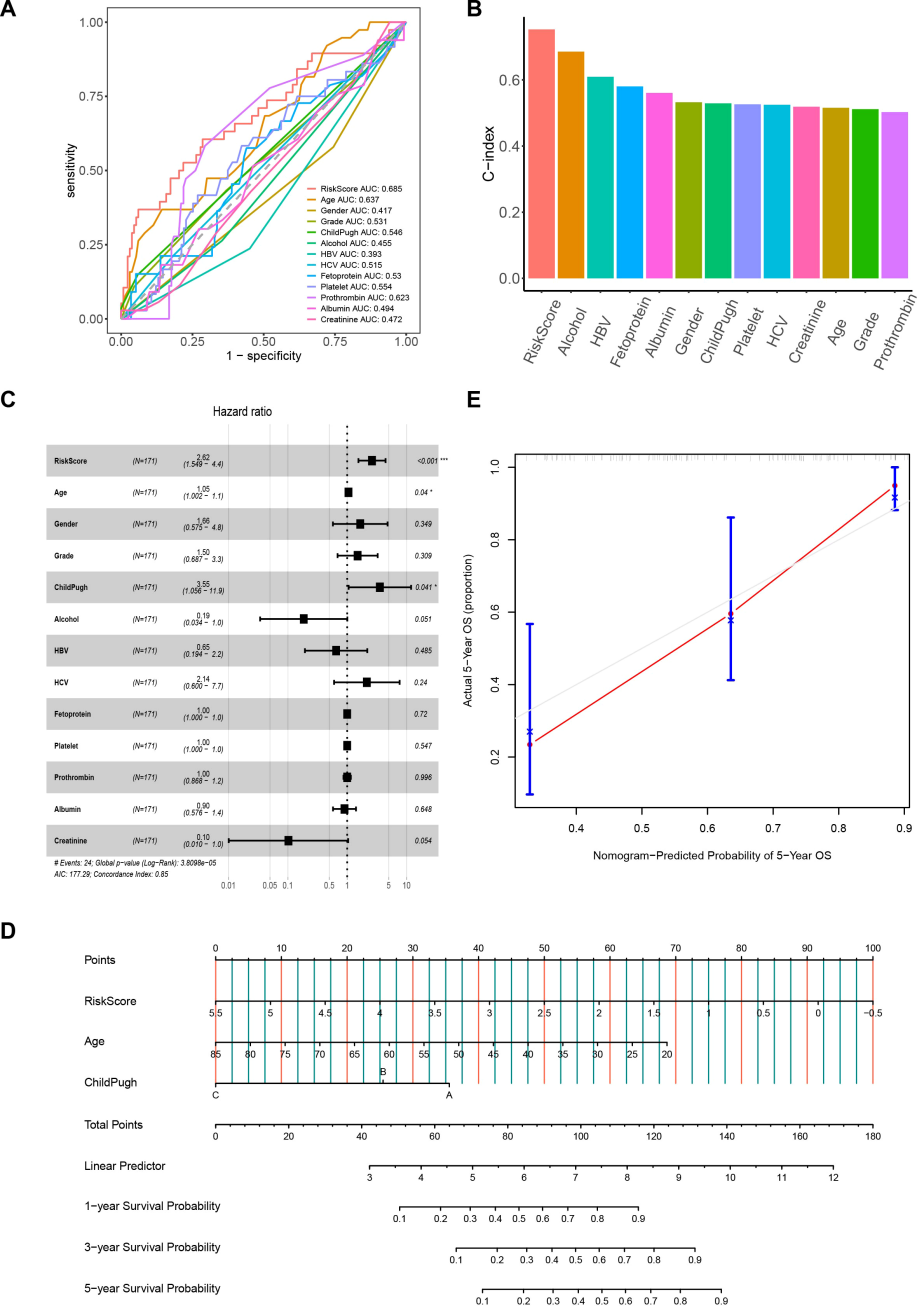
The correlation between the risk score and clinical characteristics. **(A)** ROC curves for the prognostic model and clinical characteristics. **(B)** C-index histograms for the prognostic model with different clinical characteristics. **(C)** Results of multivariate Cox regression analysis of the prognostic model and clinical characteristics. **(D)** Nomogram model created from the prognostic model, age, and Child-Pugh classification. **(E)** Five-year calibration curves. ROC, Receiver Operating Characteristic. *P< 0.05; ***P< 0.001.

To investigate the associations between the risk score and clinicopathological characteristics, we analyzed the correlations between the expression of 7 MVI-related genes and clinical parameters in HCC patients (**Supplementary Figure 4A**). The results of the Wilcoxon test demonstrated a significant correlation between the risk score and different Grade levels. As the grade level increased, the risk score also increased. However, among other clinical characteristics, the risk score was not significant (**Supplementary Figure 4B**). Furthermore, we compared the AUC values and C-index of the risk score with those of various clinical characteristics. We found that the risk score had the highest AUC and C-index, indicating better predictive performance compared to individual clinical characteristics (**Figures 4A, B**). This suggest that the prognosis model has good predictive capability. To determine whether the risk score is an independent risk factor for the prognosis of HCC patients, we also conducted a multivariate Cox regression analysis of the risk score and clinical characteristics. The results showed that the risk score, age, and Child-Pugh classification are related to the overall survival of HCC patients and can serve as independent risk factors, with the risk score being more closely associated with poor prognosis (p < 0.001) (**Figure 4C**). To make further specific predictions about individual prognosis, we integrated these independent prognostic factors to create a nomogram model. By calculating the score of each variable according to the patient’s condition and summing them to get a total score. It is possible to predict the patient’s 1-year, 3-year and 5-year survival rates, allowing an intuitive assessment of the patient’s prognosis and expanding its clinical applicability (**Figure 4D**). The calibration curve showed that the predicted 5-year survival rate was highly consistent with the actual survival rate, indicating that the nomogram is accurate and reliable (**Figure 4E**).

### 3.4 The HCC prognosis model based on MVI characteristic genes possesses good predictive value

**Figure 5:**
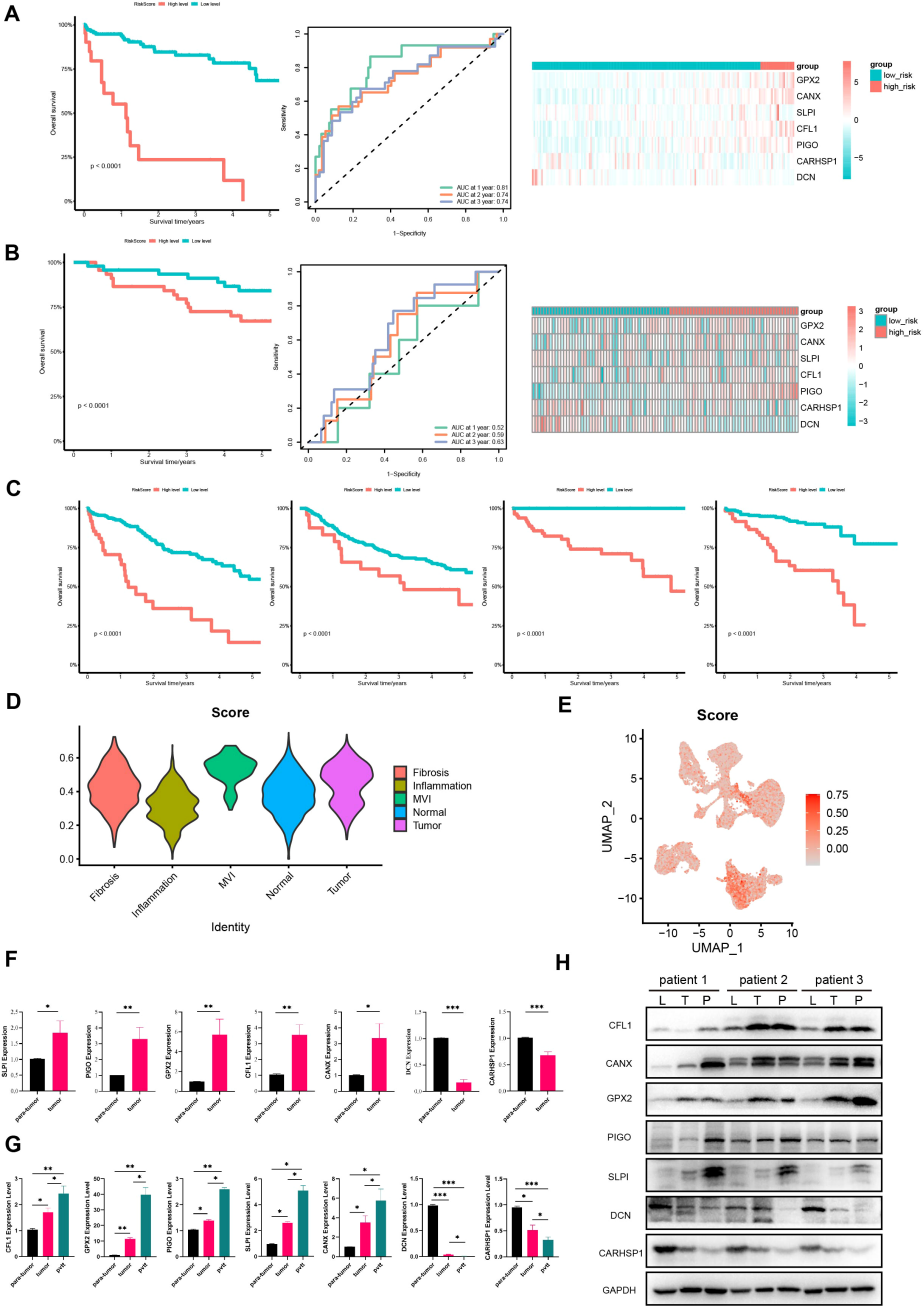
Further validation of the model in TCGA, GEO, ICGC cohorts and HCC tissues. Survival curves, ROC curves and heatmaps of model gene expression for early HCC patients from **(A)** TCGA and **(B)** GSE14520 datasets. **(C)** Survival curves for HCC patients from TCGA, GSE14520, GSE76427, and LIRI-JP databases. **(D)** Violin plots of expression of prognostic model gene set in different regions in the spatial transcriptome. **(E)** The UAMP plot of prognostic model gene set in single-nucleus transcriptomics. **(F)** The expression of protective genes and risk genes in para-tumor and tumor tissue samples. **(G)** The expression of protective genes and risk genes in para-tumor, tumor and portal vein tumor thrombus tissue samples. **(H)** Immunoblotting of proteins in the prognostic model expression (CFL1, PIGO, GPX2, SLPI, CANX, DCN, CARHSP1) in para-tumor, tumor, and portal vein tumor thrombus tissues samples. L, para-tumor; T, tumor; P, pvtt. *P <*0.05 is considered statistically significant. GEO, Gene Expression Omnibus; ICGC, International Cancer Genome Consortium. (*P < 0.05; **P < 0.01; ***P < 0.001).

To evaluate this prognostic model, we calculated the risk score of HCC patients in the TCGA cohort based on the prognostic model and classified the patients into low- and high-risk groups. The results indicated that the high-risk group had shorter survival times, higher mortality rates, and poorer prognosis compared to the low-risk group. The ROC curve demonstrated that the AUC values for 1-year, 2-year, and 3-year predictions were 0.81, 0.74, and 0.74, respectively, indicating that the model has good predictive ability for the prognosis of HCC patients (**Figure 5A**).

To further confirm the predictive value of the prognostic model in patients with HCC, we applied the same method to classify HCC patients into high- and low-risk groups in the validation cohort GSE14520. The results showed that the high-risk group in the validation cohort had shorter survival times, indicating a worse prognosis (**Figure 5B**), which is consistent with the above results. Moreover, the AUC values for 1-year, 2-year, and 3-year predictions in GSE14520 were 0.52, 0.59 and 0.63, respectively. These findings confirm the good predictive value of the prognostic model and its utility in assessing the survival risk of patients with HCC (**Figure 5B**).

In addition, we calculated the risk scores for HCC patients at all stages in the TCGA cohort and divided them into high- and low-risk groups. It turned out that the high-risk group had a worse prognosis. This was also validated in the external validation sets GSE76427 and LIRI-JP, indicating that this prognostic model can be used to predict the prognosis of HCC patients (**Figure 5C**). We also validated the expression of model genes using spatial transcriptomics. We found that the gene set of this prognostic model scored highest in the MVI regions of the spatial transcriptome (**Figure 5D**). Single-cell nuclear transcriptome UAMP plots showed the highest prognostic model gene set scores in malignant cells, validating the expression of model genes at the single-cell level (**Figure 5E**). Overall, this model can be used to predict the prognosis of HCC patients and has good predictive value.

Subsequently, we collected clinical tissues from HCC patients at our hospital and assessed the expression of 7 genes in 24 pairs of tumor and adjacent normal HCC tissues using qPCR. The results showed that protective genes (DCN and CARHSP1) were expressed at lower levels in tumors than in paired adjacent normal tissues (**Figure 5F**); risk genes (PIGO, GPX2, CFL1, SLPI, CANX) were expressed at higher levels in tumors than in paired adjacent normal tissues (**Figure 5F**). Moreover, we have also added qPCR and Western blot validation in samples of portal vein tumor thrombus. We examined the expression of these seven genes in seven pairs of para-tumor, tumor, and portal vein tumor thrombus (pvtt) tissues. The qPCR results indicated that the expression of protective genes (DCN and CARHSP1) gradually decreased in para-tumor, tumor, and pvtt tissues. Conversely, the expression of risk genes (PIGO, GPX2, CFL1, SLPI, CANX) gradually increased in these tissues (**Figure 5G**). In addition, we extracted proteins from three pairs of para-tumor, tumor, and pvtt tissues and performed western blotting (WB) experiments. The WB results showed that the expression of protective genes (DCN and CARHSP1) gradually decreased in para-tumor, tumor, and pvtt tissues. In contrast, the expression of risk-associated genes (PIGO, GPX2, CFL1, SLPI, and CANX) gradually increased in these tissues (**Figure 5H**). Therefore, these experiments further validated our prognostic model.

### 3.5 The model developed in this study outperforms other HCC models in terms of prediction performance

**Figure 6:**
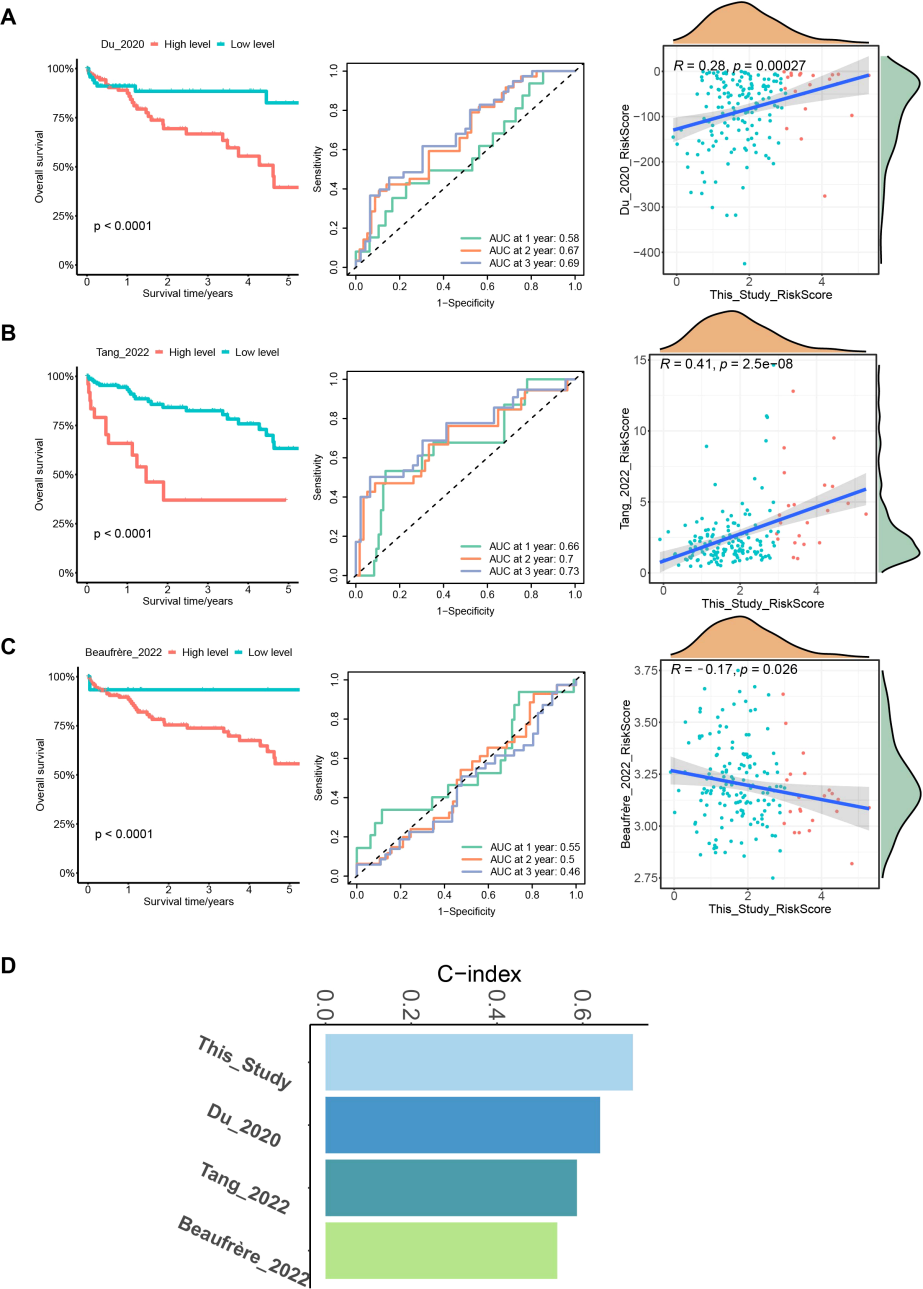
Comparison with 3 published MVI-related models. **(A)** Survival curves, ROC curves and correlation plots of risk in HCC patients predicted by the model of Du et al. compared with this model. **(B)** Survival curves, ROC curves and correlation plots of risk in HCC patients predicted by the model of Tang et al. compared with this model. **(C)** Survival curves, ROC curves and correlation plots of risk in HCC patients predicted by the model of Beaufrère et al. compared with this model. **(D)** Bar chart of C-indexes for the 4 models.

To further verify the prediction accuracy of our model, we compared it with three published HCC models. These models include a prognostic model of 7 MVI-related genes developed by Du et al. (26), a prognostic model of 3 MVI-related genes developed by Tang et al. (27) and a 6-gene HCC prediction model developed by Beaufrère et al. (28). We scored patients according to the scoring formulas provided in these three models and the ssGSEA algorithm, grouped them based on the optimal cutoff values, performed survival analysis, and plotted ROC curves (**Figures 6A–C**). We explored the correlation between our model and the three published HCC-related models using Pearson correlation analysis and considered *p* < 0.05 to indicate statistical significance. Notably, the model developed in our study showed a positive correlation with the predictive values of the models of Du and Tang et al. with consistent scoring trends (**Figures 6A, B**). However, it did not correlate with Beaufrère’s prediction model, possibly because Beaufrère et al. developed an HCC prediction model based on data obtained using NanoString technology (**Figure 6C**). Finally, we compared the corrected C-index of the four models and found that the C-index of our model was greater than that of the three published HCC-related models, which clearly shows the predictive performance of our model (**Figure 6D**).

### 3.6 The high-risk group is more susceptible to genetic mutations and immune evasion

**Figure 7:**
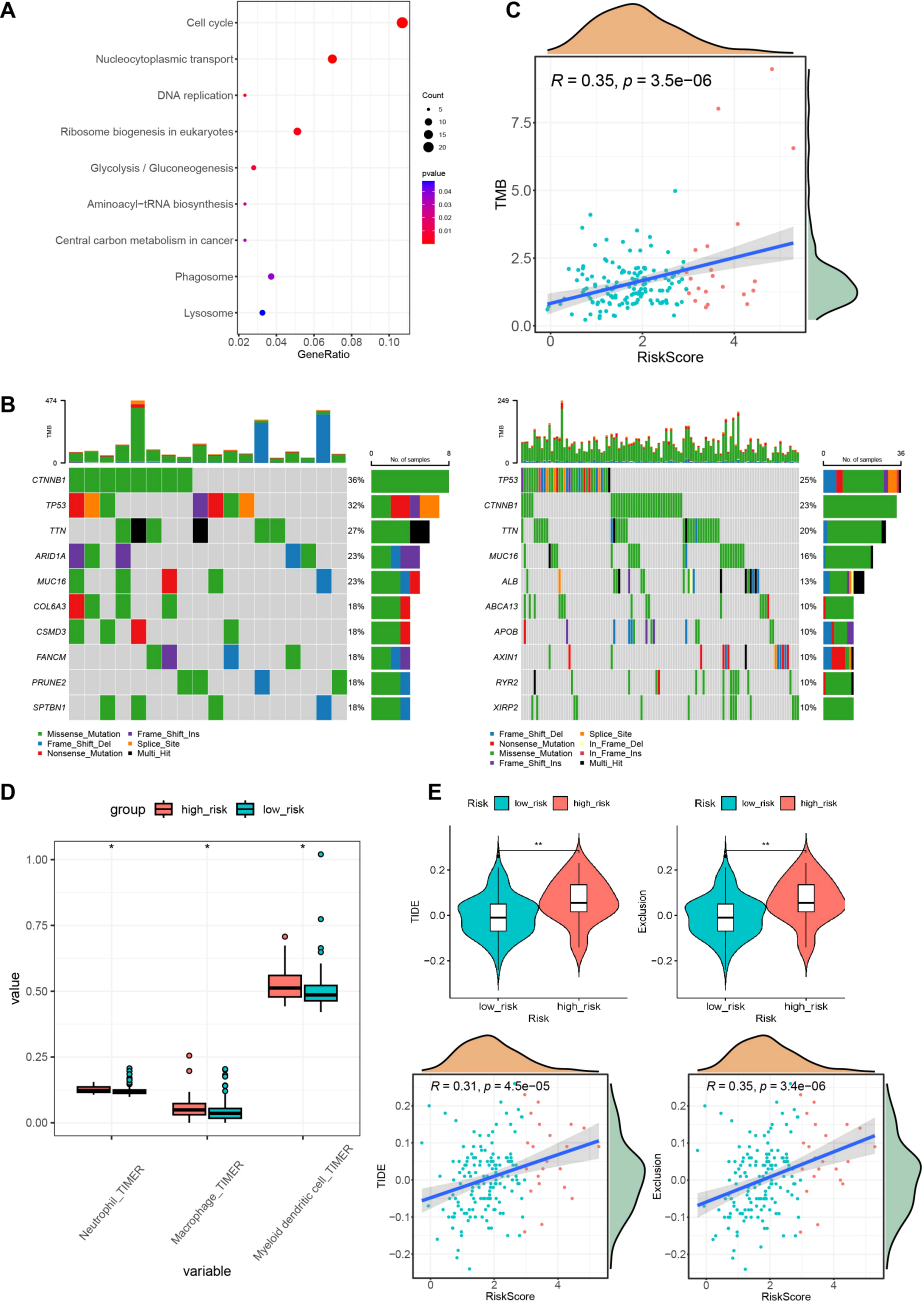
High and low-risk group mutations and immune characteristics. **(A)** KEGG enrichment results of highly expressed genes in the high-risk group. **(B)** Waterfall plots of the top 10 mutated genes in both the high- and low-risk groups. **(C)** Correlation graph between the risk score and tumor mutation burden. **(D)** Immune infiltration status in different risk groups. **(E)** TIDE scores and exclusion scores in different risk groups. KEGG, Kyoto Encyclopedia of Genes and Genomes; TIDE, Tumor Immune Dysfunction and Exclusion. *P < 0.05; **P < 0.01.

After assessing the performance of the model on various dimensions, we proceeded to evaluate the distinct characteristics of the different risk groups. Differential expression analysis was performed using the DESeq2 package for high- and low-risk groups with a threshold of FC > 2 and *p.adj* < 0.05 and identified 512 highly expressed genes in the high-risk group. KEGG pathway enrichment analysis showed that these genes were enriched in pathways related to the cell cycle, nucleocytoplasmic transport, and DNA replication (**Figure 7A**). Subsequently, the maftools package was used to analyze SNV data of HCC from TCGA. A waterfall chart was utilized to display information about the top 10 most frequently mutated genes in the high- and low-risk groups. Common mutation genes in the high-risk group, such as CTNNB1 (36% *vs*. 23%), TP53 (32% *vs*. 25%), TTN (27% *vs*. 20%) and MUC16 (23% *vs*. 16%), had higher mutation frequencies (**Figure 7B**). In addition, the TMB of early HCC patients was also calculated, which revealed a positive correlation between the risk score and tumor mutation burden (**Figure 7C**). Further investigations examined the association between the prognostic model and immune infiltration by evaluating immune cell proportions in HCC patients using TIMER 2.0. Differences in the proportions of immune cells between the high- and low-risk groups were compared. The results indicated increased proportions of macrophages, dendritic cells, and neutrophils in the high-risk group (**Figure 7D**). This suggests that these cells could promote early HCC metastasis, angiogenesis, and immune escape. TIDE was then used to predict the response of different risk groups to immunotherapy. The results demonstrated that the high-risk group had high TIDE scores and high exclusion scores and was susceptible to immune escape, resulting in worse immunotherapeutic effects (**Figure 7E**).

### 3.7 The interaction between MIF and CD74 may facilitate tumor metastasis in HCC

**Figure 8:**
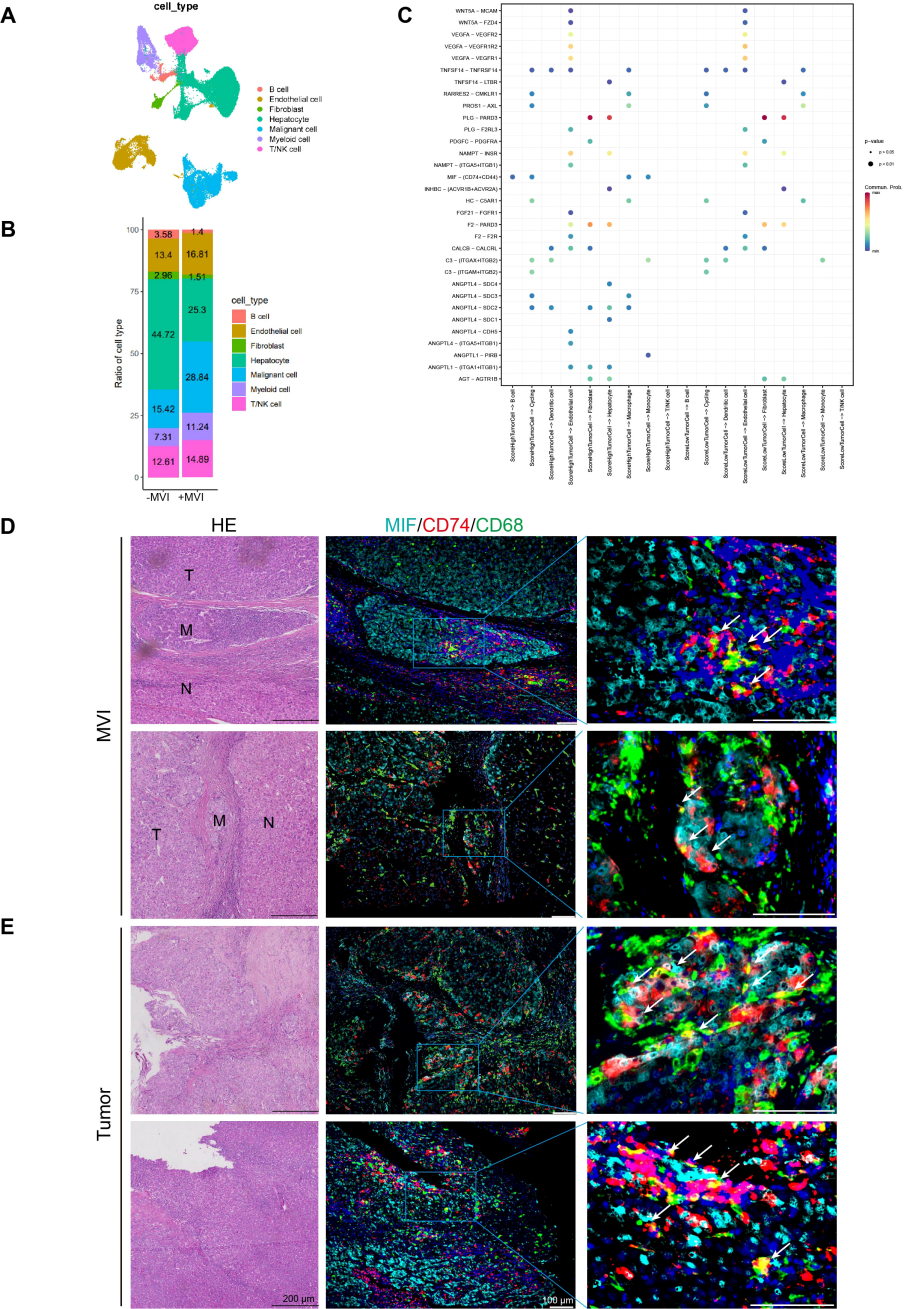
Cell interaction analysis and multiplex immunofluorescence of MVI and tumor sites. **(A)** UAMP plots of single-nuclei RNA sequencing data for various cell type markers. **(B)** Ration of cell types in MVI and no-MVI samples. **(C)** Interactions between high- and low-grade malignant cells and the receptors of other cells. **(D)** Representative HE staining and multiple immunofluorescence images of MVI sites in HCC microvascular invasion samples. **(E)** Representative HE staining and multiple immunofluorescence images of tumor sites in HCC microvascular invasion samples. UAMP, uniform manifold approximation and projection.

In the above experiments, we demonstrated that high-risk scoring patients possess a more complex immune microenvironment and are prone to immune escape. However, the mechanism of this immune evasion remains unclear. Therefore, to investigate the potential mechanisms of tumor cell immune escape and metastasis in the high-risk group, we analyzed intercellular interactions at the single-cell level. We selected 2 M0 samples and 3 MVI samples (P1_M0, P2_M0, P3_M1, P4_M2, P5_M2) for single-nucleus sequencing. The single nucleus data showed that each sample contained approximately 10,000 nuclei, with an average of 2,324 genes per cell and an average of 4,454 UMIs per cell (**Supplementary Table S5**). The quality of single-nucleus transcriptome sequencing was assessed using Cell Ranger, and after quality control, doublet removal and batch effect correction, a total of 54,771 single nuclei were obtained from the 5 samples (**Supplementary Figures 5A, B**). Then, we used the Seurat package for dimensionality reduction and clustering obtained 25 cell clusters (**Supplementary Figure 6A**). Each cluster was annotated with cell types using the singleR and scLearn packages, and copy number variations in hepatic parenchymal cells were inferred using the inferCNV package to identify normal hepatocytes and malignant cells (**Supplementary Figures 5C, D**). We identified 8 cell types: B cells, T/NK cells, myeloid cells, fibroblasts, dendritic cells, endothelial cells, normal hepatocytes, and malignant cells (**Figure 8A**; **Supplementary Figures 6B, C**). Subsequent observation of the proportions of different cell types in samples with or without MVI, it was found that the higher the degree of MVI, the higher the proportion of myeloid and T/NK cells, and that myeloid increased more (from 7.31% to 11.24%) than T/NK (from 12.61% to 14.89%) (**Figure 8B**). We further subclassified myeloid cells and identified five myeloid subpopulations: circulating cells, dendritic cells, plasma cell-like cells, monocytes, macrophages (**Supplementary Figures 7A, B**). Pathway enrichment of various myeloid subpopulations revealed that macrophages were enriched for the HIF-1 signaling pathway as well as the angiogenic pathway (**Supplementary Figures 7C, D**). Gene Set Variation analysis (GSVA) also revealed that macrophages were enriched for several signaling pathways associated with tumor progression, such as hypoxia, angiogenesis, and PI3K-AKT (**Supplementary Figure 7E**). And the Top20 gene in macrophages was associated with the prognosis of patients (**Supplementary Figure 7F**).

The ssGSEA algorithm was employed to compute prognostic model gene set scores in malignant cells from MVI samples. These malignant cells were then categorized into high and low score groups based on the median of these scores. The interactions of high- and low-scoring malignant cells with other cell types were examined using CellChat. Bar graphs showed that malignant cells with high scores had a higher number and stronger intensity of interactions with other cells (**Supplementary Figure 8A**). And heatmaps also demonstrated that high-scoring malignant cells have stronger capabilities in sending and receiving signals (**Supplementary Figure 8B**). Additionally, compared to low-scoring malignant cells, high-scoring malignant cells could communicate with macrophages, monocytes, and B cells through the MIF-(CD74+CD44) axis (**Figure 8C**). In addition intercellular interaction analysis showed that malignant cells with high and low scores had ligand-receptor interactions with macrophages but not with T/NK cells (**Figure 8C**). Therefore, we focused primarily on macrophages. Multiple immunofluorescence staining indicated that MIF-CD74 macrophages interact in the MVI region of MVI samples, where MIF-CD74 can promote tumor metastasis at the MVI site (**Figure 8D**). Furthermore, there is an interaction between MIF-CD74 macrophages in the tumor regions of the MVI samples, suggesting that their interaction may facilitate tumor progression (**Figure 8E**). However, there was no significant interaction between MIF-CD74 macrophages in the adjacent non-tumor tissues of the MVI samples and the tumor tissues of the non-MVI samples. (**Supplementary Figures 8C, D**).

The MIF-CD74 signaling pathway activates various pathways that promote cell growth and angiogenesis and inhibit the tumor suppressor protein p53 (29). Studies have shown that in kidney renal clear cell carcinoma, the strong interactions between tumor cells and tumor-associated macrophages, driven by MIF and its receptors CD74 and CD44, are critically involved in tumor progression, angiogenesis, and the mechanism of immune evasion (30). Furthermore, CD36+ cancer-associated fibroblasts (CAFs) employ MIF and CD74 to attract CD33+ myeloid-derived suppressor cells (MDSCs), creating an immunosuppressive environment that facilitates immune evasion in hepatocellular carcinoma (31). In summary, it is suggested that malignant cells in HCC could use the MIF-(CD74+CD44) interaction to promote metastasis, angiogenesis, and immune evasion.

## 4 Discussion

Hepatocellular carcinoma (HCC) is an extremely aggressive cancer and one of the most common causes of cancer-related death worldwide. It is characterized by its tendency to metastasize a high recurrence rate and considerable heterogeneity (32). Only 5%-10% of HCC patients are candidates for surgical treatment, with more than 70% experiencing recurrence within five years of surgery (1). Microvascular invasion (MVI) is considered a risk factor for postoperative recurrence and metastasis in HCC patients. Studies have shown that MVI is a predictive indicator of survival in HCC patients (28), therefore, the prediction of HCC prognosis is crucial for selecting treatment modalities and evaluating the prognosis of HCC patients. However, there are currently no accurate molecular markers for MVI to predict the prognosis of HCC patients. On this basis, we investigated different genes at MVI sites in HCC patients by spatial transcriptomics sequencing and constructed an HCC prognostic model.

In this study, we screened for differential genes at MVI sites by spatial transcriptomic sequencing. By comparing MVI locations with other regions, we identified 82 MVI-related genes. We then used early HCC data from TCGA as the training set to develop an HCC prediction model using various analytical approaches, including univariate Cox regression, LASSO regression, multivariate Cox regression, CoxBoost, random survival forests, and stepwise regression analysis. By comparing the C-index and error curves, we ultimately selected 7 key MVI genes (GPX2, CANX, SLPI, CFL1, PIGO, CARHSP1, DCN) to construct the HCC prognostic model. Studies have shown that the genes in the prognostic model influence metastasis and angiogenesis in HCC and other tumors. The expression of Glutathione Peroxidase 2 (GPX2) is associated with tumor metastasis of rat HCC both *in vitro* and *in vivo*. Reducing GPX2 expression in rat HCC cells leads to decreased migration; tail vein injection of cells with knocked down GPX2 results in reduced tumor formation capability and fewer lung metastases. Moreover, immunohistochemistry results of human HCC samples indicate that GPX2 is more highly expressed in tumor sites than in adjacent non-tumor tissues (33). High expression of GPX2 is associated with poor prognosis. Cox regression analysis shows that GPX2 expression is an independent prognostic factor for HCC overall survival. Cells with high GPX2 expression have stronger resistance to lenvatinib, making GPX2 a critical target for lenvatinib treatment in HCC (34). Calnexin (CANX) complexes on the cell surface can reduce the number of extracellular disulfide bonds, thereby degrading the extracellular matrix, which serves as a physical barrier to HCC growth, thereby inducing tumor growth and invasion (35). Secretory leukocyte peptidase inhibitor (SLPI) is upregulated in several cancer types and is highly expressed in liver cancer cell lines. Studies have shown that SLPI promotes metastasis (36). Cofilin 1(CFL1) is upregulated in the tumor tissues of HCC and is significantly associated with the overall survival and disease-free survival of HCC patients. Moreover, downregulation of CFL1 can inhibit the migration, invasion, and metastasis of HCC cells both *in vitro* and *in vivo* (37). CFL1 is also highly expressed in tumor tissues of HCC patients who are insensitive to sorafenib and is associated with poor prognosis. The co-delivery of siCFL1 and sorafenib via nanoparticles could represent a new strategy for advanced HCC (38). Furthermore, CFL1 is expressed more highly in portal vein tumor thrombus (pvtt) than in HCC tumor tissues, and an increase in CFL1 expression is closely related to adverse clinical features, making it an independent risk predictor for the overall survival of HCC patients. Silencing of CFL1 can inhibit the growth viability, invasiveness, and epithelial-mesenchymal transition (EMT) of HCC cells *in vitro*, and it can also suppress the growth and lung metastasis of HCC cells in nude mice *in vivo* (39). miR-155 influences TNF-α mRNA stability by inhibiting calcium regulated heat stable protein 1 (CARHSP1), thereby modulating the inflammatory response and protecting vessels in atherosclerosis (40). Phosphatidylinositol Glycan Anchor Biosynthesis Class O (PIGO) can serve as a potential marker for the prognosis of prostate cancer (41). Decorin (DCN) is downregulated in HCC with portal vein tumor thrombus (pvtt) tissue, and low DCN expression is associated with microvascular invasion (MVI) occurrence and poor prognosis, indicating that DCN can promote vascular invasion in HCC tissues (42). Furthermore, DCN is underexpressed in tumor tissues of HCC patients, and overexpression of DCN can inhibit the proliferation of HCC cells, while knockdown of DCN can enhance HCC cell proliferation, making it a new target for HCC (43). These studies are largely consistent with the results of our prognostic genes. Thus, these seven genes are closely related to the growth and prognosis of HCC cells, which also confirms the accuracy of modeling these seven genes to some extent.

We also carried out corresponding validations for this model. First, we performed a multivariate Cox regression analysis on risk scores and clinical characteristics. The results indicated that the risk score, age, and Child-Pugh classification were associated with the overall survival of HCC patients and served as independent risk factors. And the risk score is more closely related to a worse prognosis. We also represented independent prognostic factors in a nomogram model to visually assess patient prognosis to improve its clinical applicability. Second, we calculated the risk score for each patient and divided them into high- and low-risk groups. Survival analysis revealed that the high-risk group had shorter survival times, higher mortality rates and a worse prognosis. In addition, we collected clinical samples from HCC patients at our hospital and examined the expression of 7 genes in 24 pairs of cancerous and adjacent noncancerous HCC tissues using the qPCR assay. The results showed lower expression of DCN and CARHSP1 in tumors compared to paired adjacent noncancerous tissues; PIGO, GPX2, CFL1, SLPI, and CANX were more highly expressed in tumors than in adjacent noncancerous tissues. Furthermore, we compared the model constructed in this study with three published HCC models. The results showed that the C-index of our model exceeded that of the three published HCC-related models, which demonstrated the predictive performance of our model. These results confirm that the validations conducted further clarify the reliability and predictive value of the prognostic model and support its clinical utility for personalized treatment and prognosis prediction.

In addition, we observed the relationship between the high- and low-risk groups and the immune microenvironment. The results indicated a higher proportion of macrophages, dendritic cells, and neutrophils in the high-risk group. Moreover, the high-risk group had higher immune rejection scores. These findings suggest a more complex immune microenvironment in the high-risk group, leading to increased immune evasion and worse immunotherapy outcomes. It further confirms the importance of the prognostic model in clinical decision-making regarding treatment options for patients. Subsequently, we also explored the potential mechanisms behind HCC metastasis. We found that malignant cells can interact with macrophages through the MIF-CD74 axis, thereby promoting HCC metastasis.

The advantage of this risk scoring system is that it develops an individual scoring system for patients, where those classified as high risk have an increased probability of tumor recurrence. Additionally, this risk scoring model can predict the prognosis of early HCC patients in conjunction with age and Child-Pugh classification, and can assess the possibility of postoperative recurrence. Therefore, in the era of precision medicine, this risk evaluation model not only provides a more scientific and advanced indicator for assessing tumor recurrence and prognosis risks for clinical use but also offers guidance for personalized treatment of cancer patients.

## 5 Conclusion

To sum up, in this study, we developed and validated a prognostic model for HCC patients based on MVI genes. This model can more accurately predict the overall survival (OS) of HCC patients at different stages. Moreover, the risk score of this model can serve as an independent prognostic factor, which is of great importance for distinguishing patient types and selecting appropriate treatment options.

## Data Availability

The data presented in the study are deposited in the Genome Sequence Archive in National Genomics Data Center, China National Center for Bioinformation/Beijing Institute of Genomics, Chinese Academy of Sciences repository, accession number BioProject ID: PRJCA035412.
