# Supplementary material for: Development of a prognostic model for hepatocellular carcinoma based on microvascular invasion characteristic genes by spatial transcriptomics sequencing
Source: Front Immunol. 2025 Feb 20;16:1529569. doi: 10.3389/fimmu.2025.1529569 (PMC11882567; doi:10.3389/fimmu.2025.1529569)
Supplement: Supplementary file 1 [file DataSheet1.docx]

Development of a prognostic model for hepatocellular carcinoma based on microvascular invasion characteristic genes by spatial transcriptomics sequencing

Xiaolan Mu^1^, Lili Pan^1^, Xicheng Wang^1^, Changcheng Liu^1,2^, Yu Li^1^, Yongchao Cai^1,2,^*, Zhiying He^1,2,3,^*

^1^Institute for Regenerative Medicine, Medical Innovation Center and State Key Laboratory of Cardiology, Shanghai East Hospital, School of Life Sciences and Technology, Tongji University, Shanghai 200123, P. R. China

^2^Shanghai Engineering Research Center of Stem Cells Translational Medicine, Shanghai 200335, P. R. China

^3^Shanghai Institute of Stem Cell Research and Clinical Translation, Shanghai 200120, P. R. China

# Supplementary Methods

## Univariate Cox Regression analysis

The results of univariate Cox regression analysis are primarily used for preliminary variable selection, providing a basis for subsequent multivariate analysis and model construction. In this study, we employed the “survival” package to conduct univariate Cox proportional hazards regression analysis on genes related to the MVI site, in order to identify genes associated with prognosis.

## LASSO Regression analysis

LASSO (Least Absolute Shrinkage and Selection Operator) regression is an enhanced method of linear regression that achieves sparse variable selection and estimation of regression coefficients by incorporating an L1 regularization term into the model. Prior to conducting LASSO regression, the data are standardized so that each variable has a mean of 0 and a standard deviation of 1. In this study, we employed the “glmnet” package to perform LASSO regression analysis on potential signature genes related to the MVI site. We selected the optimal λ value in the LASSO model through 10-fold cross-validation, which corresponds to the smallest mean squared error. After determining the optimal λ, we extracted variables with non-zero coefficients from the model. These variables are considered to be significantly associated with the dependent variable. Subsequently, we identified genes with high predictive power corresponding to this λ value and further selected genes with non-zero coefficients that are related to MVI.

## CoxBoost analysis

CoxBoost is a likelihood-based boosting method used to fit the Cox proportional hazards model. It fits the model by incrementally updating the parameters of individual covariates. In this study, we used the “optimCoxBoostPenalty” function from the “CoxBoost” package to determine the penalty parameter, identifying an appropriate penalty value that ensures the optimal number of boosting steps, as determined by cross-validation, falls within the specified range. We also employed the “cv.CoxBoost” function to perform 10-fold cross-validation to determine the optimal number of boosting steps and to identify genes with non-zero coefficients that are associated with the prognosis of hepatocellular carcinoma (HCC) with microvascular invasion (MVI).

## Random Survival Forest analysis

The “randomForestSRC” package was used to perform random forest analysis. To minimize random errors as much as possible, we set the random seed from 1 to 100 to perform 100 iterations of random survival forests, and the mean minimal depth (MD) of MVI-linked genes across these iterations is calculated. Then the package rank the MVI-related genes by the average MD value. The sliding window sequential forward feature selection (SWSFS) technique is employed, which sequentially integrates each MVI-associated gene into the model. After adding each gene, the SWSFS performs a random survival forest analysis to calculate its out-of-bag error rate. This model with the lowest out-of-bag error rate is identified. The number of trees in the forest (Number of trees, ntree) that minimizes the model's error rate and stable performance is selected. A grid search is performed to determine the ideal combination of the number of variables randomly selected at each node (mtry) and the minimum number of samples at the end nodes (Nodesize) that corresponds to the lowest out-of-bag error rate. The optimal number of genes is determined. Then we calculate the average variable importance (VIMP) for MVI-related genes over 100 runs and rank the genes by this average value. Repeat the above process to determine the optimal number of genes. Finally, we compute the average VIMP and MD for MVI-related genes from 100 runs, rank the genes by average and repeat the process to find the optimal number of genes.

## Stepwise multivariate Cox Regression analysis

Finally, the selected genes are subjected to multivariate Cox regression analysis and the model is optimized through bidirectional (forward or backward) stepwise regression analysis. When the Akaike information criterion (AIC) is minimized, the model fit is considered excellent and overfitting is avoided, leading to the selection of the most prognosis-relevant genes and the formation of an HCC prognostic model. The concordance index (C-index) of each model was recalculated 999 times to select the model with the highest calibrated C-index, minimal error and best predictive performance to determine the prognosis model formula. The prognostic risk score is calculated as follows: coefficient of gene 1 × expression of gene 1 + coefficient of gene 2 × expression of gene 2 + ... + coefficient of gene n × expression of gene n.

1. **Supplementary Figure**


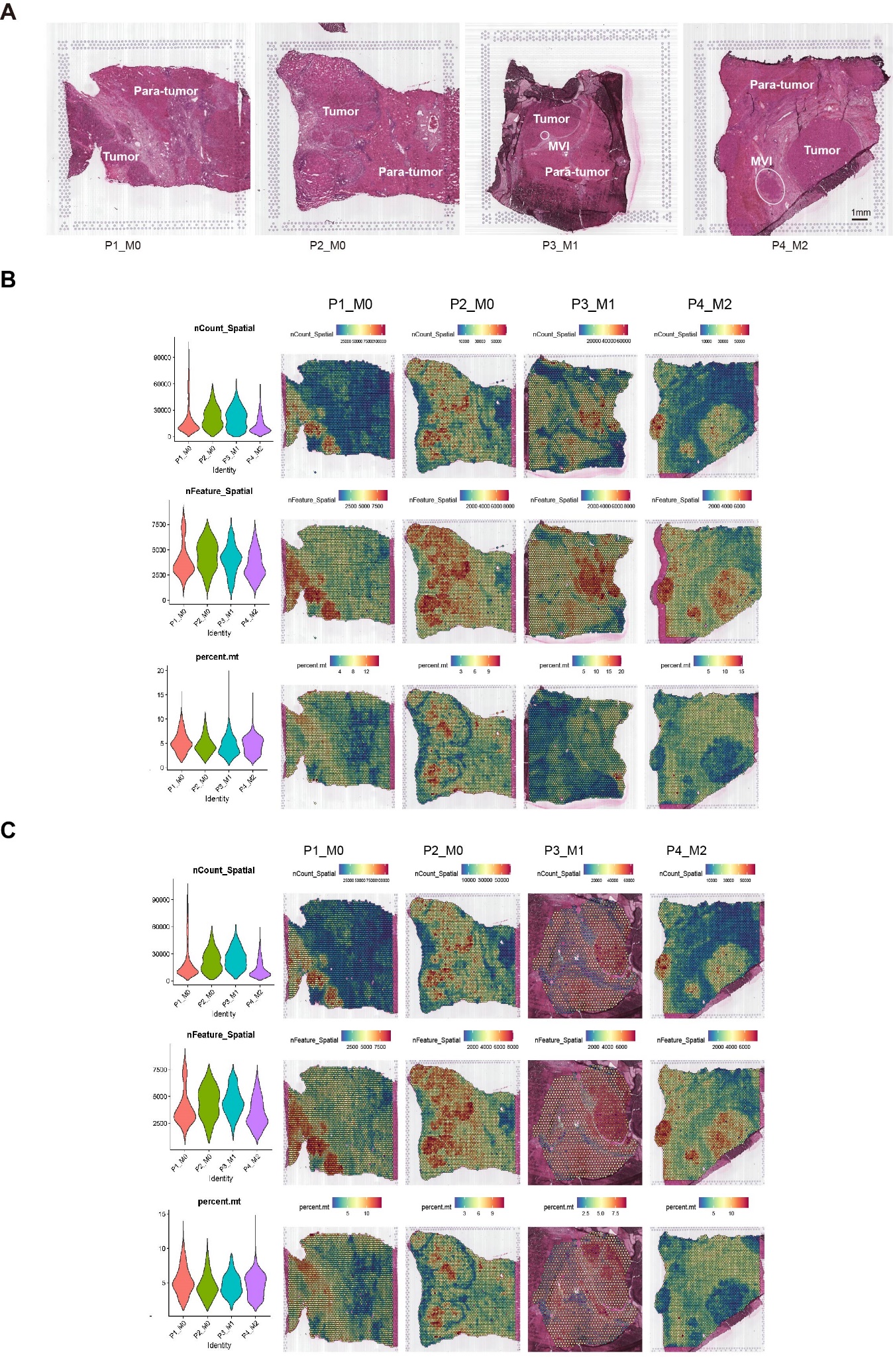


**Supplementary Figure 1. Sampling process and ST sequencing.** (A) Four tissues sections stained with H&E for spatial transcriptome sequencing. Circles indicate MVI sites. (B) Spatial feature plots of the number of expressed genes (nGene), transcripts (nUMIs) and Mitochondrial gene ratio before quality control. (C) Spatial feature plots of the number of expressed genes (nGene), transcripts (nUMIs) and Mitochondrial gene ratio after quality control. UMI, Unique Molecular Identifier.


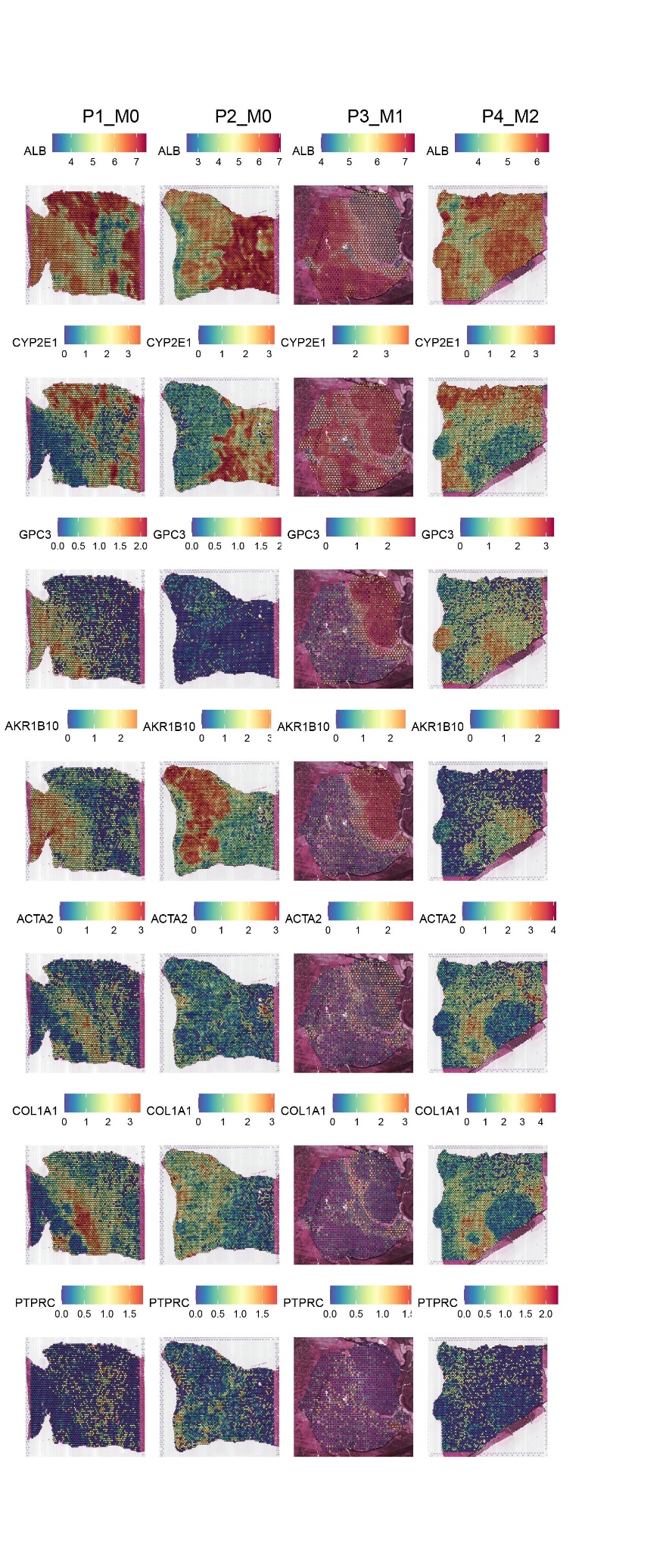


**Supplementary Figure 2. Expression of marker genes.** The spatial feature plots of seven marker genes of four tissues.


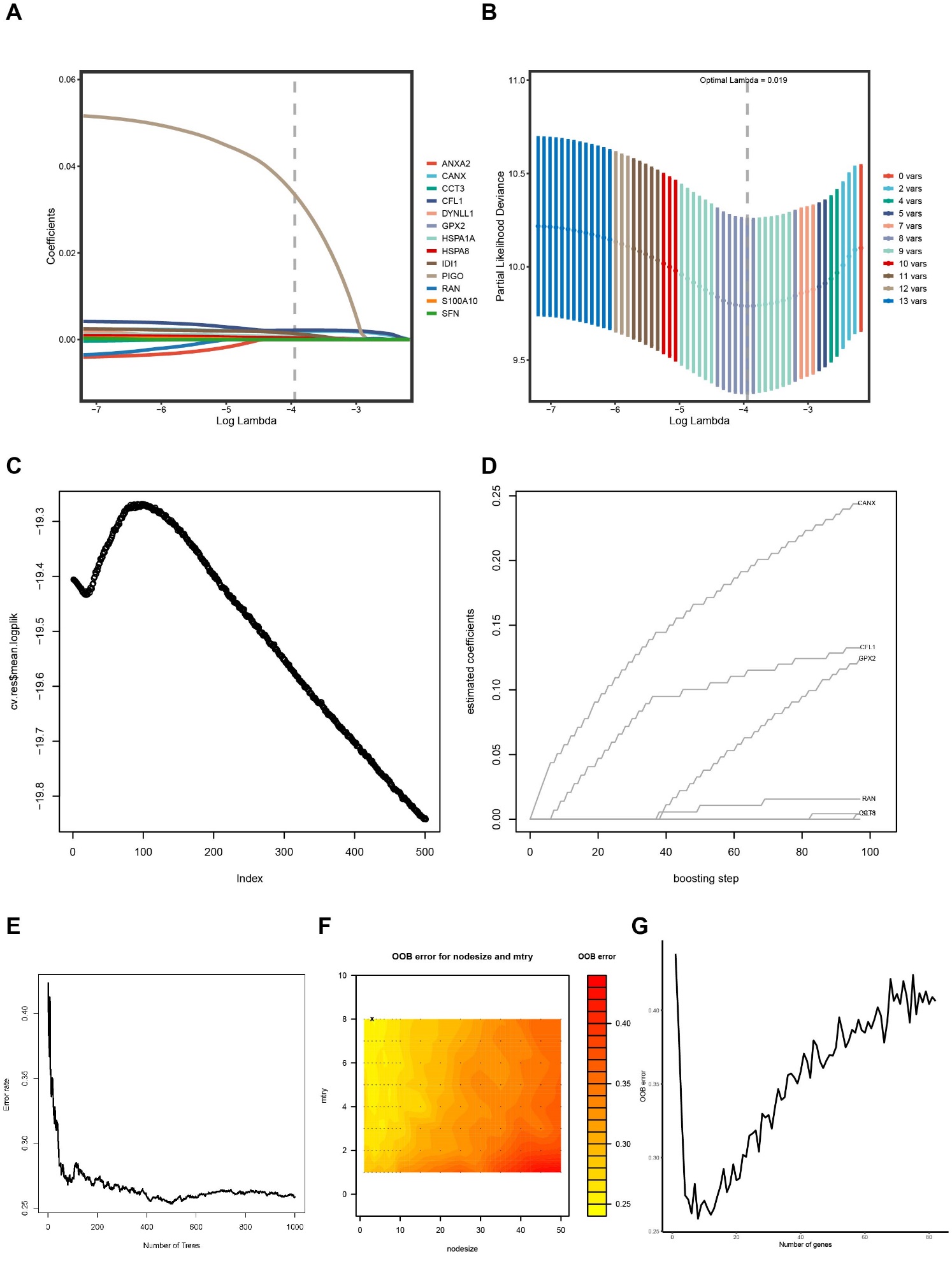


**Supplementary Figure 3. Using Univariate Cox regression, CoxBoost and random survival forest modeling methods to construct an HCC prognostic model in TCGA cohort.** (A) Coefficients of individual genes under different penalty parameters in LASSO regression analysis. (B) Number of genes under different penalty parameters in LASSO regression analysis. (C) CoxBoost analysis the relationship between different boosting steps and average local log-likelihood. (D) Coefficients of individual genes under different boosting steps in CoxBoost analysis. (E) Relationship between ntree and error rate in random survival forest analysis. (F) Error rates under different combinations of nodesize and mtry parameters. (G) Relationship between the number of genes in the model and error rate in random survival forest analysis. LASSO, Least absolute shrinkage and selection operator.


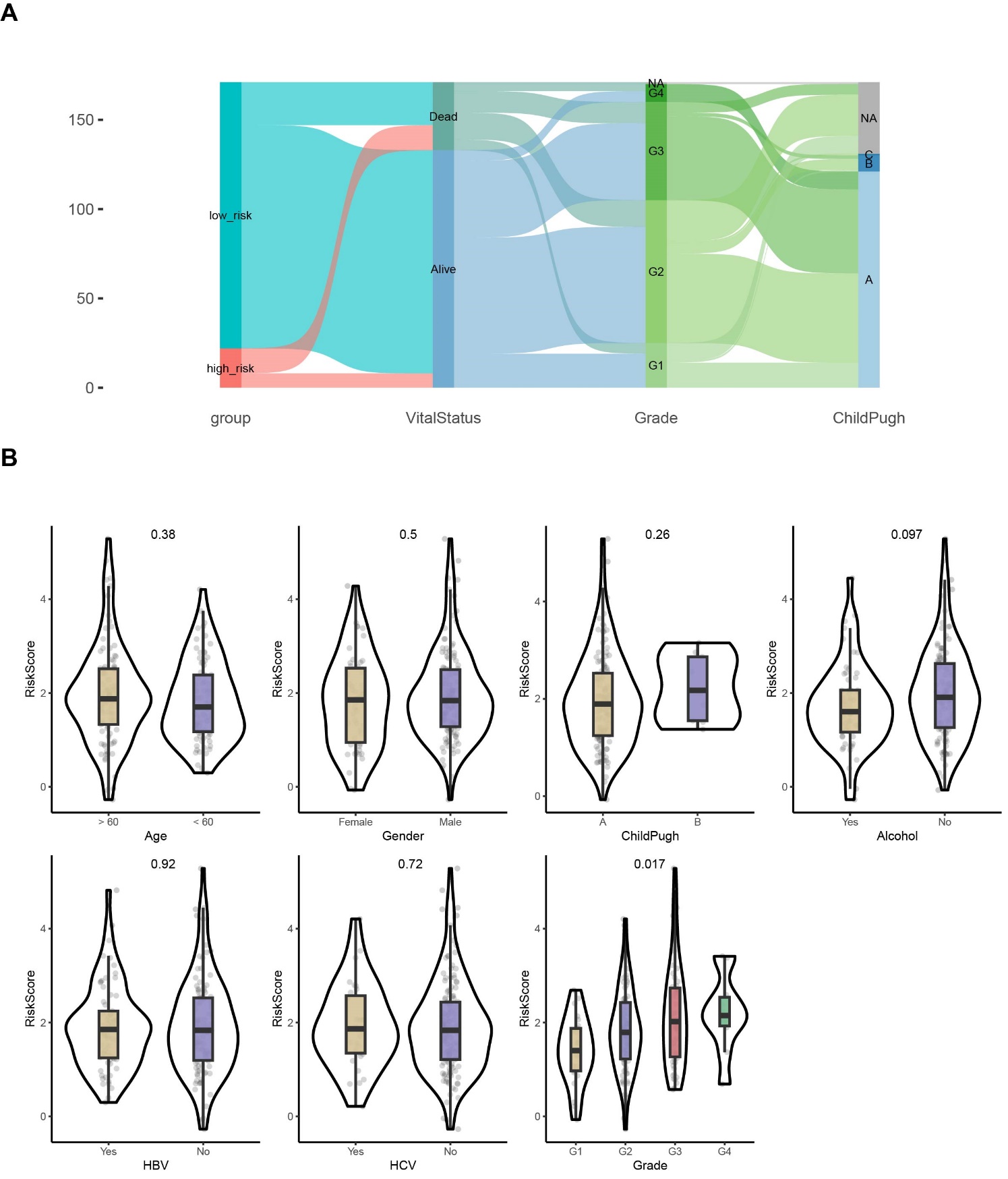


**Supplementary Figure 4. The correlation between the risk score and clinical data and subgroup analysis.** (A) Sankey diagram of risk grouping with survival status, Grade classification and ChildPugh classification. (B) Risk scoring situation of different clinical feature subgroups.


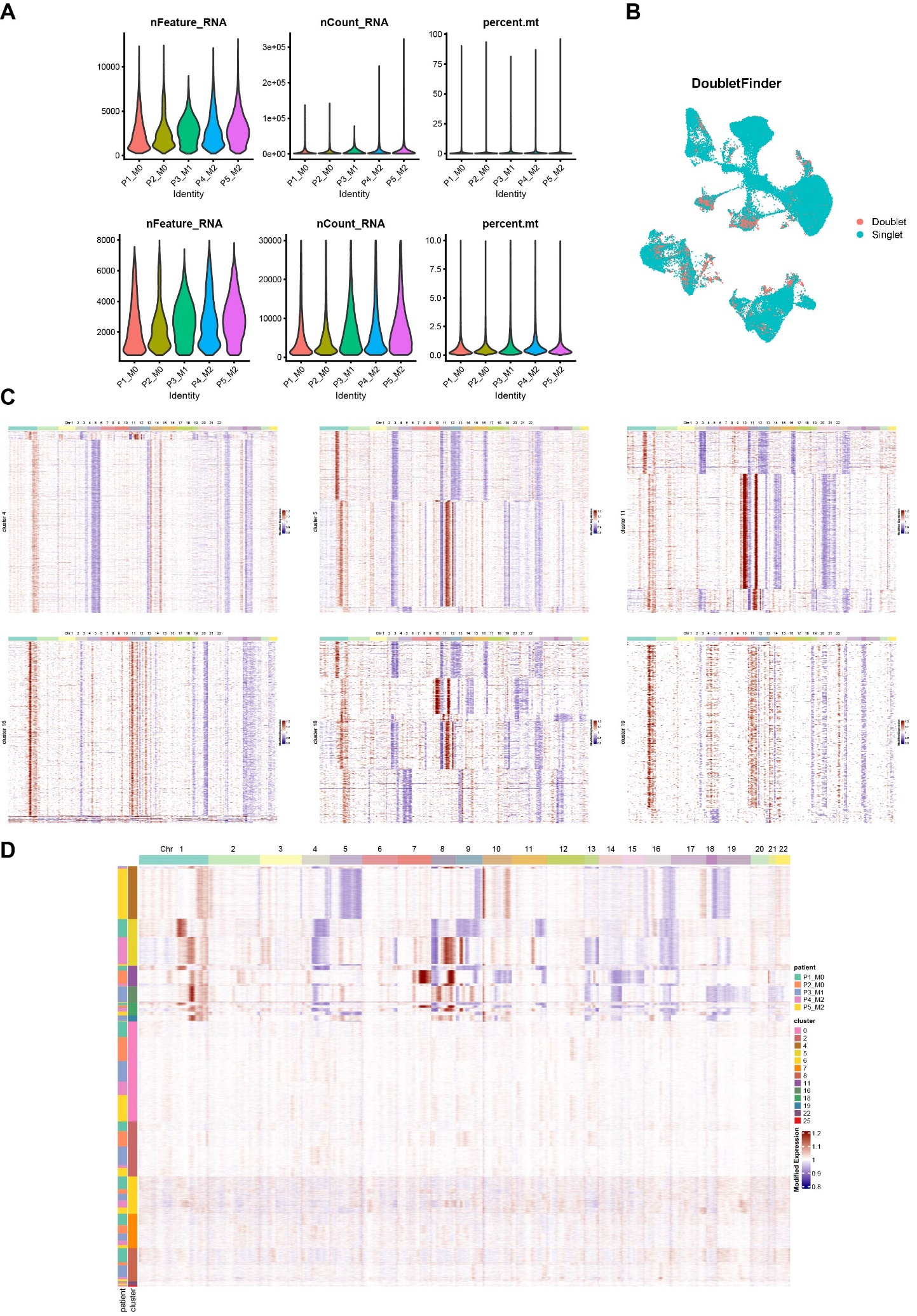


**Supplementary Figure 5. Single-nuclei RNA sequencing, quality control and Identification of benign and malignant cells.** (A) The number of expressed genes (nGene), transcripts (nUMIs) and Mitochondrial gene ratio for each patient. The top of A is before quality control and the bottom of A is after quality control. (B) Single-cell, double-cell single-nucleus transcriptome sequencing UAMP map. (C) Malignant cell cluster copy number variation graphs. (D) Different hepatocyte cluster copy number. UAMP, Uniform Manifold Approximation and Projection.


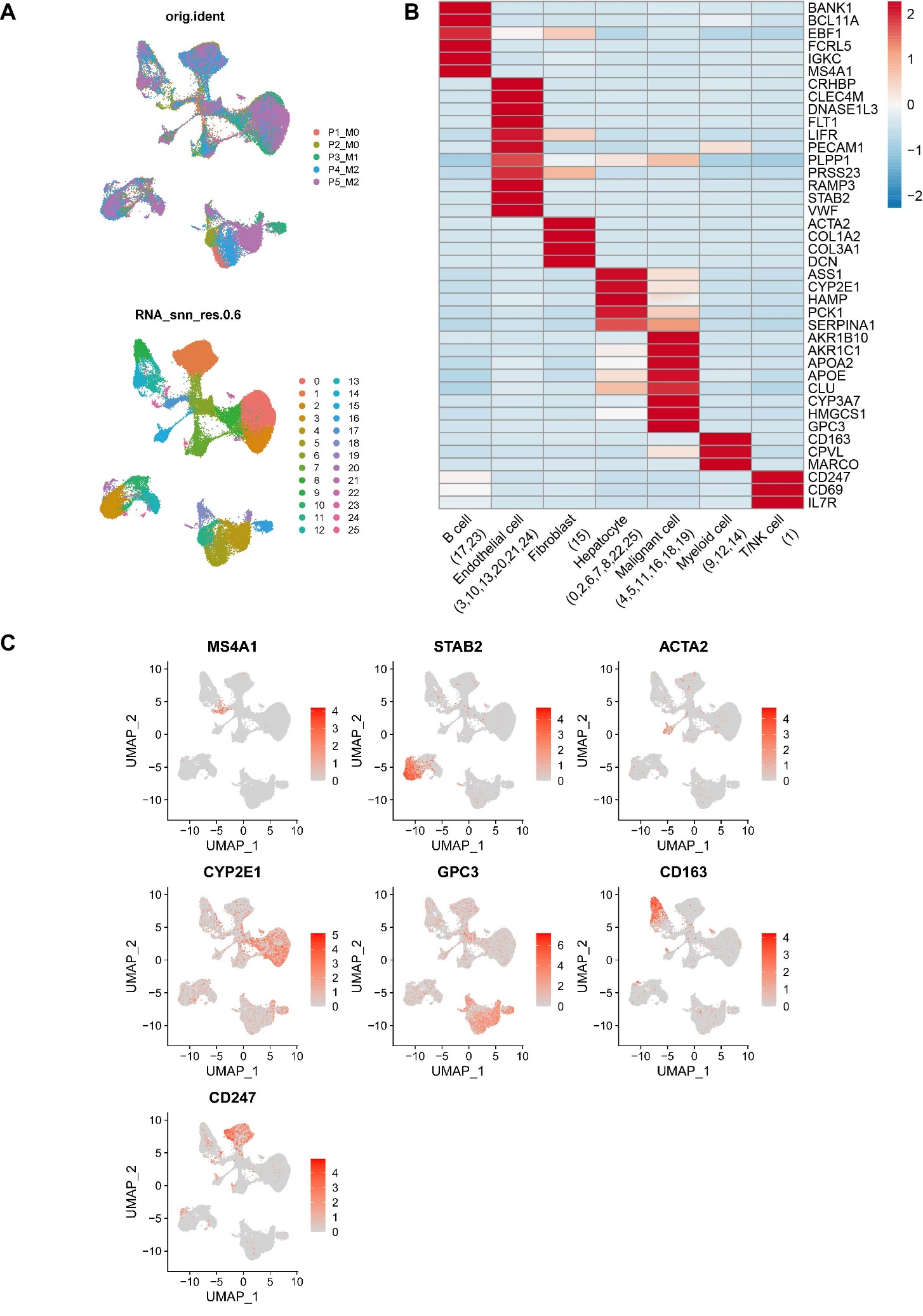


**Supplementary Figure 6. Single-nucleus data dimensionality reduction clustering.** (A) Single-nucleus transcriptome sequencing UAMP maps with patient and cell cluster markers. The top of A is patient UAMP map; the bottom of A is cell cluster markers UAMP map. (B) Heatmap of marker genes for each cell types. (C) The UAMP plots of seven marker genes of five tissues.

**
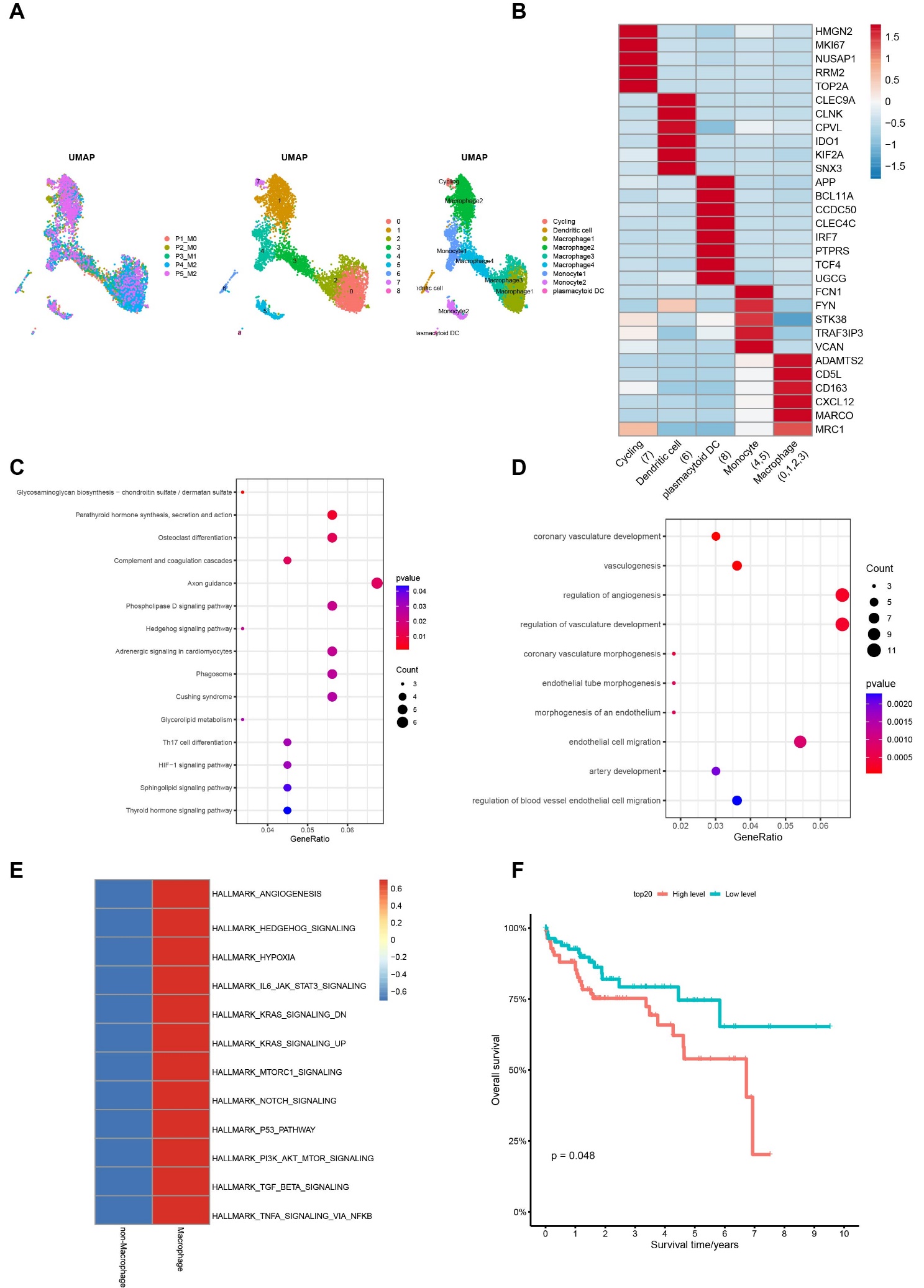
Supplementary Figure 7. Subclassification of myeloid cells and pathway enrichment analysis of macrophages.** (A) UAMP plot of myeloid cell subclassification. (B) Heat map of marker genes for various myeloid subgroups. (C) Kyoto Encyclopedia of Genes and Genomes analysis of macrophages. (D) Biological Process analysis of macrophages. (E) Gene Set Variation analysis of macrophages and non-macrophages in myeloid cells. (F) Survival analysis of the top 20 genes in macrophages.


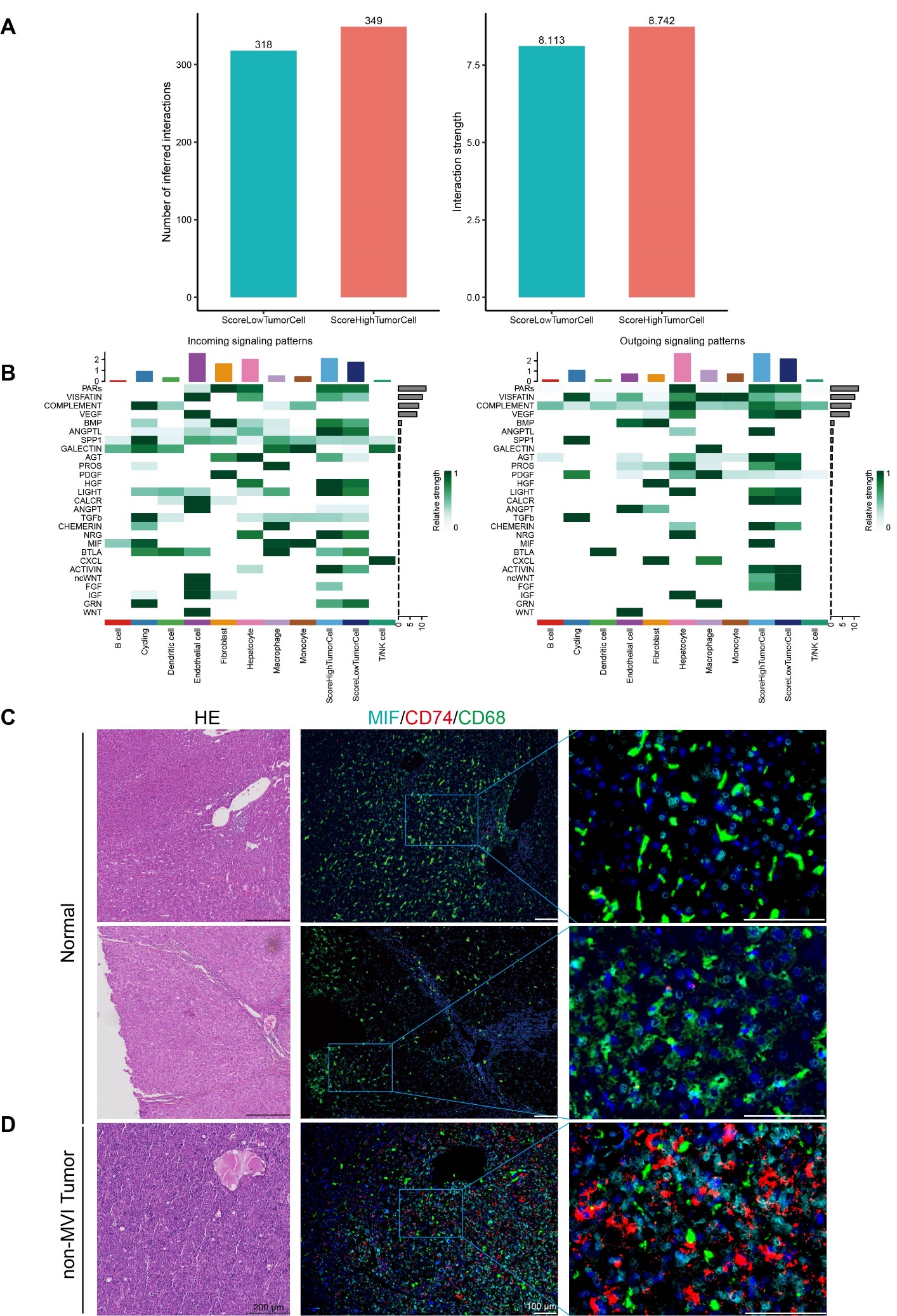


**Supplementary Figure 8. Cell interaction analysis and multiplex immunofluorescence of tumor and peri-tumoral areas.** (A) High and low scores of malignant cell interaction numbers and intensities. (B) The ability of various cells to receive and send signals. (C) Representative HE staining and multiplex immunofluorescence at adjacent normal tissues of MVI samples. (D) Representative HE staining and multiplex immunofluorescence at tumor tissue of non-MVI sample.
