# Supplementary material for: Development of a prognostic model for hepatocellular carcinoma based on microvascular invasion characteristic genes by spatial transcriptomics sequencing
Source: Front Immunol. 2025 Feb 20;16:1529569. doi: 10.3389/fimmu.2025.1529569 (PMC11882567; doi:10.3389/fimmu.2025.1529569)
Supplement: Supplementary file 2 [file DataSheet2.docx]

######################################CoxBoost##########################################

##########

library(randomForestSRC)

library(CoxBoost)

days <- dat$OS

status <- dat$VitalStatus

x <- as.matrix(dat[,-(1:3)])

##########

set.seed(seed)

optim.res <- optimCoxBoostPenalty(time = days,

status = status,

x = x,

trace = FALSE,

start.penalty = 10)

optim.res$penalty

##########

set.seed(seed)

cv.res <- cv.CoxBoost(time = days,

status = status,

x = x,

maxstepno = 500,

K = 10,

type = "verweij",

penalty = optim.res$penalty)

cv.res$optimal.step

##########

cbfit <- CoxBoost(time = days,

status = status,

x = x,

stepno = cv.res$optimal.step,

penalty = optim.res$penalty)

summary(cbfit)

value <- cv.res$optimal.step+1

names <- cbfit$xnames[which(cbfit$coefficients[value,]!=0)]

coef <- cbfit$coefficients[value,][which(cbfit$coefficients[value,]!=0)]

outTab <- as.data.frame(cbind(names,coef))

outTab$coef <- as.numeric(outTab$coef)

colnames(outTab) <- c("GeneSymbol","Coefficient")

##########

names <- outTab$GeneSymbol

names <- c("OS","VitalStatus",names)

subExp <- dat[,names]

multiCox=coxph(Surv(OS, VitalStatus) ~ ., data = subExp)

multiCox=step(multiCox, direction="both")#backward、forward

multiCoxSum=summary(multiCox)

multiCoxSum

outTab=data.frame()

outTab=cbind(

Coefficient=multiCoxSum$coefficients[,"coef"],

HR=multiCoxSum$conf.int[,"exp(coef)"],

HR.95L=multiCoxSum$conf.int[,"lower .95"],

HR.95H=multiCoxSum$conf.int[,"upper .95"],

Pvalue=multiCoxSum$coefficients[,"Pr(>|z|)"])

outTab=as.data.frame(cbind(GeneSymbol=row.names(outTab),outTab))

outTab[,2:6] <- apply(outTab[,2:6],2,as.numeric)

length(outTab$GeneSymbol)

outTab$GeneSymbol

######################################LASSO##########################################

##########

library(survival)

pFilter=0.05

outTab=data.frame()

sigGenes=c("OS","VitalStatus")

for(gene in colnames(dat[,4:ncol(dat)])){

cox=coxph(Surv(OS, VitalStatus) ~ dat[,gene], data = dat)

coxSummary = summary(cox)

coxP=coxSummary$coefficients[,"Pr(>|z|)"]

if(coxP<pFilter){

sigGenes=c(sigGenes,gene)

outTab=rbind(outTab,

cbind(GeneSymbol=gene,

HR=coxSummary$conf.int[,"exp(coef)"],

HR.95L=coxSummary$conf.int[,"lower .95"],

HR.95H=coxSummary$conf.int[,"upper .95"],

Pvalue=coxP) )

}

}

outTab[,2:5] <- apply(outTab[,2:5],2,as.numeric)

table(outTab$Pvalue < 0.05)

outTab[outTab$Pvalue < 0.05,]$GeneSymbol

surSigExp=dat[,sigGenes]

surSigExp=cbind(id=row.names(surSigExp),surSigExp)

##########

library(survival)

library(survminer)

library(glmnet)

surSigExp$OS[surSigExp$OS<=0]=0.003

x=as.matrix(surSigExp[,c(4:ncol(surSigExp))])

y=as.matrix(Surv(surSigExp$OS,surSigExp$VitalStatus))

fit <- glmnet(x, y, family = "cox", maxit = 1000)

cvfit <- cv.glmnet(x, y, family="cox", maxit = 1000)

coef <- coef(fit, s=cvfit$lambda.min)

index <- which(coef != 0)

lassoGene=row.names(coef)[index]

length(lassoGene)

lassoGene

lassoGene=c("OS","VitalStatus",lassoGene)

lassoSigExp=surSigExp[,lassoGene]

lassoSigExp=cbind(id=row.names(lassoSigExp),lassoSigExp)

##########

lassoSigExp$id <- NULL

multiCox=coxph(Surv(OS, VitalStatus) ~ ., data = lassoSigExp)

multiCox=step(multiCox, direction="both")#backward or forward

multiCoxSum=summary(multiCox)

multiCoxSum

outTab=data.frame()

outTab=cbind(

Coefficient=multiCoxSum$coefficients[,"coef"],

HR=multiCoxSum$conf.int[,"exp(coef)"],

HR.95L=multiCoxSum$conf.int[,"lower .95"],

HR.95H=multiCoxSum$conf.int[,"upper .95"],

Pvalue=multiCoxSum$coefficients[,"Pr(>|z|)"])

outTab=as.data.frame(cbind(GeneSymbol=row.names(outTab),outTab))

outTab[,2:6] <- apply(outTab[,2:6],2,as.numeric)

length(outTab$GeneSymbol)

outTab$GeneSymbol

######################################RSF##########################################

##########

library(survival)

library(survminer)

library(randomForestSRC)

library(timeROC)

library(data.table)

library(tidyr)

library(dplyr)

library(tibble)

library(caret)

library(ggRandomForests)

all <- list()

for(i in 1:100){

print(i)

seed <- seed+i

set.seed(seed)

rsf_t <- rfsrc(Surv(OS,VitalStatus)~.,data = dat,

ntree = 1000,

splitrule = 'logrank',

importance = T,

proximity = T,

forest = T,

block.size = 1,

seed = seed)

varsel_pbc <- var.select(rsf_t)

gg_md <- gg_minimal_depth(varsel_pbc)

var <- gg_md$varselect

var <- var[order(var$depth),]

var$depth_order <- 1:num

var <- var[order(-var$vimp),]

var$vimp_order <- 1:num

all[[i]] <- var

if(i == 1){

ord <- var[,3:5]

colnames(ord)[2:3] <- c("depth.1","vimp.1")

} else{

var[,1:2] <- NULL

ord <- merge(ord,var,by = "names")

colnames(ord)[2*i] <- paste("depth",i,sep = ".")

colnames(ord)[2*i+1] <- paste("vimp",i,sep = ".")

}

}

rownames(ord) <- ord$names

ord$names <- NULL

depth <- ord[,seq(1,ncol(ord),2)]

vimp <- ord[,seq(0,ncol(ord),2)]

ord.mean <- data.frame(apply(ord[,seq(1,ncol(ord),2)],1,mean),apply(ord[,seq(0,ncol(ord),2)],1,mean))

ord.mean$mean <- apply(ord,1,mean)

ord$depth.mean <- ord.mean[,1]

ord$vimp.mean <- ord.mean[,2]

ord$mean <- ord.mean[,3]

ord <- ord[order(ord$vimp.mean),]

ord$VIMP.Rank <- 1:num

ord <- ord[order(ord$depth.mean),]

ord$Depth.Rank <- 1:num

ord$names <- rownames(ord)

ord$names <- factor(ord$names,levels = ord$names)

ord <- ord[order(ord$mean),]#vimp.mean,mean

ord$Rank <- 1:num

colname <- c("OS","VitalStatus",as.character(ord$names))

dat <- dat[,colname]

##########

rsf <- list()

OOB.error <- list()

for(i in 3:dim(dat)[2]){

print(i)

if(i == 3){

formula <- as.formula(paste0('Surv(OS, VitalStatus)~',colnames(dat)[3]))

} else{

formula <- as.formula(paste0('Surv(OS, VitalStatus)~', paste(colnames(dat[,3:i]), sep = '', collapse = '+')))

}

o <- tune(formula,dat[,1:i],ntreeTry = 1000)

rsf_i <- rfsrc(Surv(OS,VitalStatus)~.,data = dat[,1:i],

ntree = 1000,

nodesize = o$optimal[1],

mtry = o$optimal[2],

splitrule = 'logrank',

importance = T,

proximity = T,

forest = T,

block.size = 1,

seed = seed+101)

rsf[[i-2]] <- rsf_i

OOB.error[[i-2]] <- tail(rsf_i$err.rate,1)

}

##########

error <- as.data.frame(unlist(OOB.error))

error$number <- 1:nrow(error)

colnames(error)[1] <- "OOB.error"

number <- which(error==min(error),arr.ind=TRUE)[1]

colnumber <- number+2

dat_best <- dat[,1:colnumber]

rsf_best <- rsf[[number]]

gene_best <- colnames(dat[,3:colnumber])

##########

formula <- as.formula(paste0('Surv(OS, VitalStatus)~', paste(colnames(dat_best[,3:dim(dat_best)[2]]), sep = '', collapse = '+')))

o <- tune(formula,dat_best,ntreeTry = 1000)

##########

multiCox=coxph(Surv(OS, VitalStatus) ~ ., data = dat_best)

multiCox

multiCox=step(multiCox, direction="both")#backward or forward

multiCox

multiCoxSum=summary(multiCox)

multiCoxSum

outTab=data.frame()

outTab=cbind(

Coefficient=multiCoxSum$coefficients[,"coef"],

HR=multiCoxSum$conf.int[,"exp(coef)"],

HR.95L=multiCoxSum$conf.int[,"lower .95"],

HR.95H=multiCoxSum$conf.int[,"upper .95"],

Pvalue=multiCoxSum$coefficients[,"Pr(>|z|)"])

outTab=as.data.frame(cbind(GeneSymbol=row.names(outTab),outTab))

outTab[,2:dim(outTab)[2]] <- apply(outTab[,2:dim(outTab)[2]],2,as.numeric)

length(outTab$GeneSymbol)

outTab$GeneSymbol
